# *Buddleja globosa* Leaf Methanolic Extract Acts Against *Trypanosoma cruzi* Parasites by Inducing Mitochondrial Inner Membrane Hyperpolarization

**DOI:** 10.3390/plants14172749

**Published:** 2025-09-02

**Authors:** Helena Quintero-Pertuz, Vicente Valenzuela-Bass, Michel Lapier, José Ortega-Campos, Sebastián Alfaro, Gilsane Lino von Poser, Christian Espinosa-Bustos, Adriano Costa de Camargo, Fabiola González-Herrera, Juan D. Maya, Raquel Bridi

**Affiliations:** 1 Departamento de Química Farmacológica y Toxicológica, Facultad de Ciencias Químicas y Farmacéuticas, Universidad de Chile, Dr. Carlos Lorca Tobar 964, Independencia, Santiago 8380492, Chile; helena.quintero@ug.uchile.cl (H.Q.-P.); v.valenzuela.4@ug.uchile.cl (V.V.-B.); 2 Grupo de Investigación Fitotécnia del Trópico, Universidad del Magdalena, Carrera 32 No 22–08, Santa Marta 47001, Colombia; 3 Centro de Investigación, Desarrollo e Innovación de Productos Bioactivos (CinBio), Escuela de Química y Farmacia, Facultad de Farmacia, Universidad de Valparaíso, Av. Gran Bretaña 1093, Valparaíso 2360102, Chile; michel.lapier@uv.cl; 4 Laboratorio de Radicales Libres y Antioxidantes, Facultad de Ciencias Químicas y Farmacéuticas, Universidad de Chile, Dr. Carlos Lorca Tobar 964, Independencia, Santiago 8380494, Chile; jose.ortega.c@uchile.cl; 5 Programa de Farmacología Molecular y Clínica, Instituto de Ciencias Biomédicas, Facultad de Medicina, Universidad de Chile, Avenida Independencia 1027, Independencia, Santiago 8380453, Chile; sebalfarolobos@gmail.com; 6 Programa de Pós-Graduação em Ciências Farmacêuticas, Laboratório de Farmacognosia, Faculdade de Farmácia, Universidade Federal do Rio Grande do Sul, Porto Alegre 90610-000, RS, Brazil; gilsane.von@ufrgs.br; 7 Departamento de Farmacia, Facultad de Química y de Farmacia, Pontificia Universidad Católica de Chile, Santiago 702843, Chile; ccespino@uc.cl; 8 Nutrition and Food Technology Institute, University of Chile, Santiago 7830490, Chile; adrianoesalq@gmail.com; 9 Programa de Inmunología, Instituto de Ciencias Biomédicas (ICBM), Facultad de Medicina, Universidad de Chile, Avda. Independencia 1027, Block I, 3er piso, Santiago 8380453, Chile; fabiola.gonzalez@ug.uchile.cl

**Keywords:** *Buddleja globosa*, *Trypanosoma cruzi*, Chagas disease, catalpol, natural products

## Abstract

The neglected Chagas disease, a zoonosis caused by the *Trypanosoma cruzi* parasite, has limited treatment options like nifurtimox and benznidazole, known for their toxic effects and controversial efficacy. Natural products present opportunities for therapeutic alternatives, particularly in Chile, which has a rich variety of endemic flora. This study focused on the Chilean *Buddleja globosa*, evaluating the antioxidant activities and biological effects of its methanolic extract (MET) and BG500, an enriched iridoid fraction (6-*O*-methylcatalpol), against *T. cruzi* trypomastigotes. Although the trypanocidal activity of the extract was significantly lower than that of nifurtimox (280 ± 3.5 vs. 5.0 ± 0.5), its selectivity was comparable (selectivity index > 15). The MET and enriched fraction also induced hyperpolarization of mitochondrial membrane potential (ΔΨm). In silico docking studies suggested that *T. cruzi*’s Old Yellow (OYE) could be a potential target for 6-*O*-methylcatalpol. This work provides the first report on the potential trypanocidal activity of a *B. globosa* extract, highlighting the need for further studies to connect ΔΨm and OYE inhibition to the effects of 6-*O*-methylcatalpol.

## 1. Introduction

Chagas disease (CD) is a vector-borne Neglected Zoonotic Disease caused by a flagellate protozoan, *Trypanosoma cruzi* (*T. cruzi*), that affects various mammalian species across America, including humans and domestic animals. However, due to increased population movements, *T. cruzi* infection is considered a worldwide health concern and is no longer restricted to endemic countries [1]. According to recent data, CD has an annual incidence of 30,000 new cases in 21 Latin American countries, affecting nearly 6 million people and causing, on average, 12,000 deaths annually [2]. Furthermore, an estimated 8600 newborns become infected during gestation [3]. Globally, CD creates an annual burden exceeding 800,000 disability-adjusted life years and USD 600 million in healthcare costs [4]. Currently, the infection is treated with nifurtimox (NFX, Lampit^®^, Bayer (Whippany, NJ, USA), (*RS*)-*N*-(3-metil-1,1-dioxo-1,4-tiazinan-4-il)-1-(5-nitro-2-furil)metanimine) and benznidazole (BNZ), first manufactured by Roche (Roche 7-501, Rochagan^®^, *N*-benzyl-2-(2-nitro-1*H*-imidazol-1-yl) acetamide, Basel, Switzerland). BNZ is available in the United States after being approved by the US Food and Drug Administration in 2018. Since the early 1970s, patients have been receiving the same NFX- or BNZ-based treatments, which are long-lasting and induce mild-to-moderate toxic effects in adults [5,6]. It is effective in recently infected people and controversially effective in the chronic phase, where the level of evidence for this recommendation is only moderate [7]. Furthermore, reports indicate that naturally resistant strains may limit the effectiveness of benznidazole and nifurtimox [8,9]. No new treatments have been developed, although imidazole derivatives, such as posaconazole, showed promise in preclinical studies but did not achieve the expected results in clinical trials [10,11,12]. A set of very promising benzoxaborole derivatives has been successful in non-human primates with naturally acquired CD [13]. Clinical studies are underway, although tested against *T. brucei* [14]. Thus, there is an urgent need to find new compounds with trypanocidal activity that strengthen therapeutic alternatives for treating CD in all its stages, in which the use of natural products is an open field of intense research.

Indeed, screening natural products can provide broad structural chemical diversity and offer significant opportunities for finding novel active principles [15,16].

Chile is a country with a wide range of geographic features and climates, leading to a unique variety of native plants. About 50% of these plants are considered endemic, meaning they are only found in this region [17]. Additionally, Chile is home to valuable ancestral knowledge held by its native peoples, who have a deep understanding of nature that has been passed down through generations [18]. In response to this rich natural heritage, the Chilean Ministry of Health took a significant step by certifying medicinal plants as part of its national drug policy. This certification is outlined in the Regulation of the National System of Control of Pharmaceutical Products for Human Use (DS N°3/2010) [19]. The goal is to recognize and promote the use of these plants as complementary options for improving health.

The single mitochondria of Trypanosomatids is an attractive trypanocidal target, as it produces Reactive Oxygen Species (ROS) and relies on the trypanothione/trypanothione reductase system, along with other oxidoreductases like the Old Yellow Enzyme (OYE), an NADPH oxidoreductase unique to *T. cruzi* that has a wide variety of substrates, including nitro esters, nitroaromatics, or α,β-unsaturated compounds [20,21]. These parasites, unlike their hosts, have limited ROS scavenging capabilities, a feature that can be exploited for the development of new therapies [22]. Various natural compounds and extracts show potential in targeting parasite mitochondria, for example, polyphenols like quercetin from *Hypericum afrum* Lam. [23], flavonoids from *Delphinium staphisagria* L. [24], and kaempferol derivatives from *Nectandra oppositifolia* Nees & Mart. [25]. Other effective compounds include kaurenoic acid from *Castanedia santamartensis* R.M. King & H. Rob. [26] and sesquiterpenoids from Chilean plants like *Drimys winteri* J.R. Forst. & G. Forst., *Podanthus mitiqui* Lindl., and *Maytenus boaria* Molina [27], which alter mitochondrial membrane potential and contribute to their antiparasitic effects.

*Buddleja globosa* Hoppe (Scrophulariaceae), also known as matico, stands out among the many Chilean traditional medicinal plants because of its wound and gastric ulcer healing properties [28]. Commonly used in Chile, Argentina, Peru, and Bolivia, it is particularly significant in Mapuche and Aymara cultures, serving as a healing agent [28]. Matico is applied in various ways, including poultices for wounds and infusions for treating stomach ulcers, scabies, and syphilis [28]. Research on its alcoholic extracts has identified components like saponins, terpenes, flavonoids, and iridoids [28,29,30].

Iridoids are important secondary metabolites found in a wide variety of species of medicinal plants in the Lamiales order [31]. Phytochemical studies show that iridoids are major bioactive compounds with potent activities, including anti-inflammatory, antioxidant, neuroprotective, cardioprotective, antitumor, and antibacterial effects. Notable iridoids from *B. globosa* include catalpol and aucubin [32,33]. Catalpol exhibits multiple benefits, such as leishmanicidal properties [34], antioxidative and anti-inflammatory effects, and protection against osteoporosis and knee osteoarthritis [35,36,37]. It also increases mitochondrial membrane potential in liver cells [38], but its effects on *T. cruzi* remain unstudied.

Therefore, this study evaluated the trypanocidal activity of commercially available catalpol, the methanolic extract of *B. globosa* (MET), and its fraction (BG500), enriched in an iridoid, against *T. cruzi* trypomastigotes. We hypothesized that iridoids from *B. globosa* could induce the death of *T. cruzi* by affecting mitochondrial membrane potential. In addition, the possible contribution of other compounds in MET was investigated. In parallel, an in silico assay was performed to guide the identification of potential molecular targets that predicted NADPH oxidoreductase Old Yellow Enzyme (OYE) as a promising target in the parasite.

## 2. Results

### 2.1. NMR Characterization of the Iridoid Present in the BG500 Fraction

**Figure 1:**
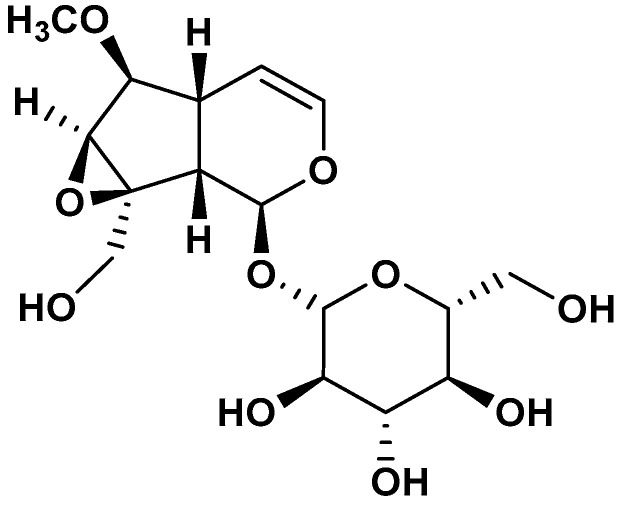
^1^H NMR chemical shifts of 6*-O-*methylcatalpol.

We searched for iridoids in the ten fractions from MET using thin-layer chromatography. Only in the BG500 fraction was the presence of an iridoid detected. The nuclear magnetic resonance data of the BG500 fraction are presented in Table 1 and Figure 1.

The ^1^H and ^13^C NMR data of the iridoid found in BG500 are shown in Table 1 and Figure 1. The ^1^H-NMR spectrum (400 MHz; CD_3_OD) (Figure S1) exhibited signals for δH 6.36 (d, *J* = 5.9 Hz, H-3), δH 5.07 (d, *J* = 9.7 Hz, H-1), δH 5.03 (m, H-4), δH 4.16 (d, *J* = 13.1 Hz), and 3.82 (d, *J* = 13.1 Hz) for methylenic protons at C-10, δH 3.68 (b.s, H-7), δH 3.65 (m, H-6), δH 3.50 (s, OCH_3_), δH 2.54 (t, *J* = 8.1 Hz, H-9), and δH 2.35 (m, H-5). Additionally, sugar core protons were observed. The doublet at δH 4.79 (d, *J* = 7.8 Hz) corresponds to the proton of the anomeric carbon. The coupling constant indicates the β configuration of the glucopyranose unit linked to the iridoid scaffold. The ^13^C-NMR (Figure S2) and DEPT spectra (101 MHz; CD_3_OD) (Figure S3) revealed the signals for δC 140.58 (C-3), δC 102.59 (C-4), δC 93.81 (C-1), δC 87.15 (C-6), δC 65.01 (C-8), δC 57.77 (C-7), δC 56.62 (OCH_3_), δC 41.83 (C-9), and δC 35.96 (C-5), corresponding to the carbons of the iridoid core.

Upon analyzing the available spectral data, a resemblance to the spectra previously published for catalpol, a compound already reported in *B. globosa*, can be observed [39]. However, in addition to the signal for a methoxy group, a notable difference is found in the ^13^C NMR concerning the chemical shift value of carbon 6, which appears downfield with a variation of approximately 10 ppm in the current iridoid.

A comparison of the ^1^H and ^13^C NMR, as well as the ^1^H-^1^H COSY (Figures S4 and S5) and HMBC spectral data of the iridoid, indicates very similar signal patterns to those of 6*-O-*methylcatalpol, reported by Hamedi et al. [40].

### 2.2. Antioxidant Activity and Polyphenol Identification and Quantification

#### 2.2.1. Antioxidant Activity of MET and Fractions

It is known that methanolic extracts from Scrophulariaceae plants contain various chemical compounds often associated with antioxidant activity [41]. To evaluate the antioxidant content and capacity of the MET and its various fractions, we quantified the total polyphenol and flavonoid content and assessed their antioxidant capacity. These results are summarized in Table 2. However, we did not evaluate the flavonoid content of the BG300 and BG400 fractions due to sample depletion.

As shown in Table 2, there are statistically significant differences between the MET and its fractions for all of the performed assays (*p* < 0.0001, according to one-way ANOVA analysis and Tukey’s post-test; see Tables S1–S10). MET values were higher for three methods (FRAP, DPPH, and TPC) than those of their fractions (Table 2). The BG400 fraction exhibited the most significant antioxidant activity, as determined by means of the ABTS assay. The MET and BG600 fractions followed this. In the DPPH assay, the MET fraction exhibited the most significant activity, followed by the BG600 fraction. These results suggest that compounds with the highest bioactivity are present in these intermediate polarity fractions (Table 2).

The most active fraction in the TPC assay was BG600, followed by BG400. However, in the TFC assay, the most active fraction was BG200, suggesting that flavones and flavonols that react with AlCl_3_ are probably not responsible for most of MET’s antioxidant activity (Table 2).

#### 2.2.2. Polyphenol Identification and Quantification

To determine if the antioxidant properties were due to iridoids and other antioxidants, such as polyphenols, a UPLC-ESI-MS/MS analysis was performed on the MET and BG500 fraction to identify the present phenolic compounds. The results are presented in Table 3. Eight polyphenolic compounds were identified, including rutin, quercetin, and caffeic acid. MET has higher levels of caffeic acid and luteolin, while quercetin levels remain consistent between the two fractions. Notably absent from the BG500 fraction are rutin and chlorogenic acid.

### 2.3. In Vitro Cytotoxic Activity of MET and the BG500 Fraction

Table 4 shows that the MET fraction BG500 exhibited trypanocidal activity with an IC_50_ value of 358 ± 4.2, while the MET fraction exhibited an IC_50_ value of 280 ± 3.5. Additionally, all evaluated samples presented a selectivity index (SI) greater than 15. Nifurtimox was used as a positive control. Notably, although the potency of MET and the BG500 fraction was approximately two orders of magnitude lower than nifurtimox, their selectivity was similar.

### 2.4. Effect of MET and Catalpol on ΔΨm

It is well known that polyphenols affect the ΔΨm independently of their typical antioxidant properties [42]. Therefore, we investigated the effects of MET, BG500 fraction, and catalpol on the ΔΨm of *T. cruzi* by using the specific probe TMRM and measuring intracellular calcium flux.

**Figure 2:**
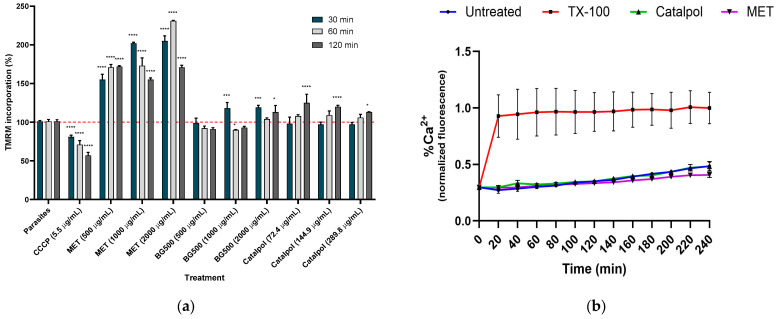
(**a**) Effect of the MET, BG500 fraction, and catalpol on *T. cruzi* ΔΨm. Trypanosomes were exposed to CCCP, MET, BG500 fraction, or catalpol for 2 h. CCCP was used as a depolarization control. The values represent the mean ± SD of triplicate determinations from three independent experiments. Statistical analysis was performed using two-way ANOVA, followed by Dunnett’s multiple comparison test. * *p* = 0.05, *** *p* < 0.001, **** *p* < 0.0001 vs. untreated parasites (details in Figures S9 and S10 and Tables S11 and S12). (**b**) Intracellular Ca^2+^ levels in *T. cruzi* trypomastigotes treated with MET (500 μg/mL), BG500 (500 μg/mL), or catalpol (72.4 µg/mL) for 4 h. Untreated and 0.5% TX-100-treated trypomastigotes were controls. Fluorescence is reported as the percentage relative to time 0. Statistical analysis was performed using ANOVA followed by Dunnett’s multiple comparison test, *** *p* < 0.0001. The values correspond to the three independent experiments’ mean ± SD of triplicate determinations.

Figure 2a shows that the MET extract significantly hyperpolarized the ΔΨm, reaching 231.1% (95% CI: 229.4–231.7; CV: 0.21%) after 60 min of incubation at a concentration of 2000 µg/mL, compared to control parasites with a mean of 100.9% (95% CI: 94.7–107.0%; CV: 2.46%). This effect depends on concentration and time, the time effect being more noticeable at a concentration of 500 µg/mL (two-way ANOVA, *p* < 0.0001).

In contrast, the hyperpolarization caused by 1000 µg/mL of BG500 was more pronounced after 60 and 120 min of incubation (*p* < 0.0002 and *p* < 0.0006, respectively) than the control, though still less than that observed with MET. Changes in ΔΨm caused by catalpol were comparable to those induced by BG500 (*p* > 0.9999), particularly at the highest concentrations. However, it is important to note that MET, BG500, and catalpol did not affect intracellular calcium flux (Figure 2b).

### 2.5. Docking Study on T. cruzi Oxidoreductases

To correlate the observed mitochondrial hyperpolarization and the antioxidant effects of MET and 6*-O-*methylcatalpol, an in silico docking study was performed on four enzymes that present oxidoreductase activity (Table 5).

Of all of the trypanosomatid oxidoreductases analyzed, OYE had the lowest binding energy (Table 5). Therefore, it was chosen for further docking analyses.

A redocking model was generated between the enzyme and flavin mononucleotide (FMN), one of its natural ligands, to model the active site of the OYE enzyme, allowing for the identification and comparison of the main interactions with the docking of 6*-O-*methylcatalpol (root-mean-square deviation (RMSD) 2.0 Å). Comparative redocking using Maestro provided an RMSD of 4.36 Å. A hydrophobic contact is observed when both molecules are present in the enzyme’s active site (Figure S11). This effect is evident in inhibitors such as menadione [43]. Docking of OYE with 6*-O-*methylcatalpol was performed without the cofactor, and the main interactions of the compound were evaluated using the cavity method. FMN and 6*-O-*methylcatalpol exhibited maximum affinity values of −7.000 kcal/mol and −6.411 kcal/mol, respectively, for different clusters (Table S13). These differences suggest strong interactions between the ligand and protein in the in silico model.

To validate the in silico model, redocking was performed between the enzyme and flavin mononucleotide (FMN), one of the enzyme’s natural ligands, to model the active site of the OYE enzyme. A 2.0 Å RMSD suggests that the docking protocol accurately reproduced the ligand’s conformation and orientation in the active site, enabling the identification and comparison of the primary interactions between 6-*O*-methylcatalpol and the amino acids of the OYE enzyme. A hydrophobic contact was also observed when both molecules were present in the enzyme’s active site (Figure S11). This effect is evident in classic inhibitors, such as menadione [43] (Figure S11B).

Docking of the OYE enzyme with 6-*O*-methylcatalpol was performed without the cofactor, and the main interactions of the compound were evaluated using the cavity method. FMN and 6-*O*-methylcatalpol exhibited maximum affinity values of −7.000 and −6.411 kcal/mol, respectively, across different clusters (Table S13). These differences suggest strong interactions between the ligand and the protein in the in silico model.

**Figure 3:**
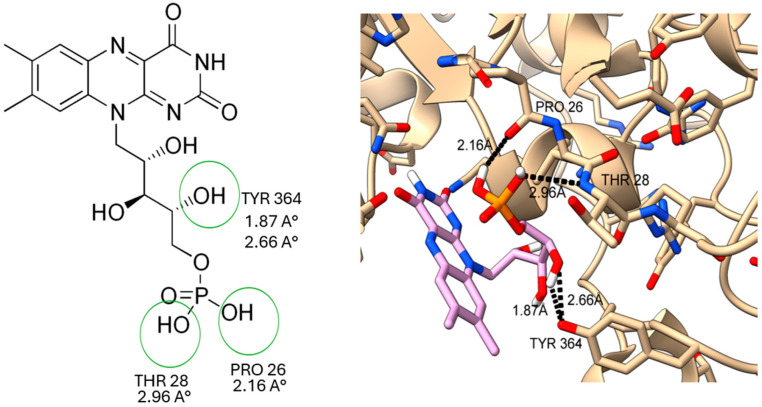
The main hydrogen bond interactions of the natural ligand FMN (redocking) with the amino acid residues of the OYE from *T. cruzi* are shown. The left panel shows the 2D structure of the FMN ligand, with green circles highlighting the chemical groups involved in hydrogen bonding with the key enzyme residues (tyrosine 364 [TYR], threonine 28 [THR], and proline 26 [PRO]), along with the corresponding distances in angstroms. The right panel shows a 3D representation of the interaction where FMN (magenta) binds to the active site of the OYE enzyme (cream). Black dashed lines indicate hydrogen bond distances, corroborating the interactions detailed in the 2D panel and highlighting the importance of these residues in stabilizing the ligand–enzyme complex.

**Figure 4:**
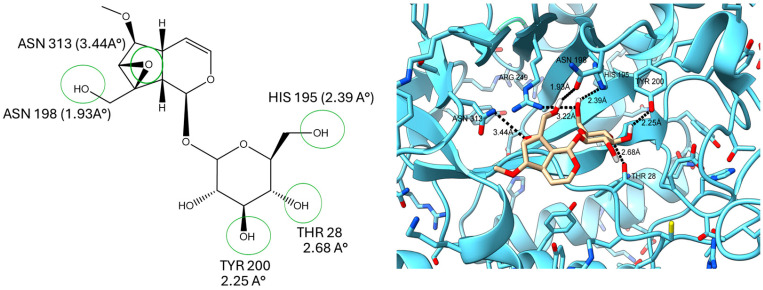
Main hydrogen bond interactions of 6*-O-*methylcatalpol with amino acid residues of OYE from *T. cruzi*. These interactions were obtained from a molecular docking study and visualized in Chimera X. The left panel shows the 2D structure of 6*-O-*methylcatalpol. Green circles highlight the hydroxyl groups involved in key hydrogen bond interactions. Specific enzyme amino acid residues (Alanine 337 (ALA), Threonine 28 (THR), and Tyrosine 200 (TYR)) and their corresponding bond distances in angstroms are indicated. The right panel shows the 3D representation of 6*-O-*methylcatalpol (beige) bound in the active site of the OYE enzyme (light blue ribbon). Black dashed lines indicate formed hydrogen bonds and their respective distances, confirming the interactions detailed in the 2D scheme and emphasizing their role in stabilizing the ligand–enzyme complex.

The results showed that the predominant interactions in ligand-receptor binding are hydrogen bonds and hydrophobic contacts. THR 28 appears to be key in ligand-protein binding, with distances of 2.96 Å and 2.68 Å for FMN and 6*-O-*methylcatalpol, respectively, as observed in models with higher affinity energy. PRO26 (2.16 Å) and TYR364 (1.87–2.66 Å), on the other hand, interact with FMN (Figure 3), while 6*-O-*methylcatalpol interacts with ASN198 (1.93 Å), HIS195 (2.39 Å), ARG249 (3.22 Å), and TYR200 (2.25 Å). Additionally, the epoxide group interacts more strongly with the protein than with residue ASN313 (3.44 Å) (Figure 4).

**Figure 5:**
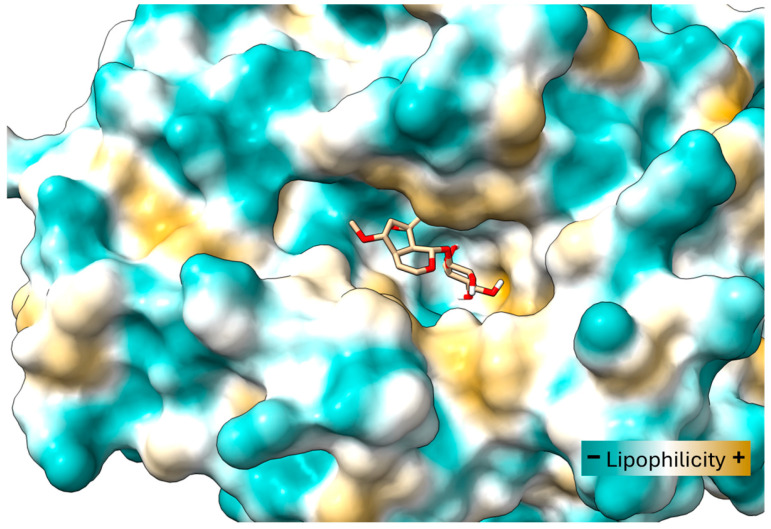
Molecular lipophilicity potential (MLP) maps for OYE and the 6*-O-*methylcatalpol ligand generated by Chimera X. The coloring on the enzyme’s molecular surface ranges from dark cyan (most hydrophilic) through white to dark goldenrod (most lipophilic). The bound ligand is shown in a stick model. This analysis highlights putative interactions between the ligand and the lipophilic pockets of the OYE enzyme.

**Figure 6:**
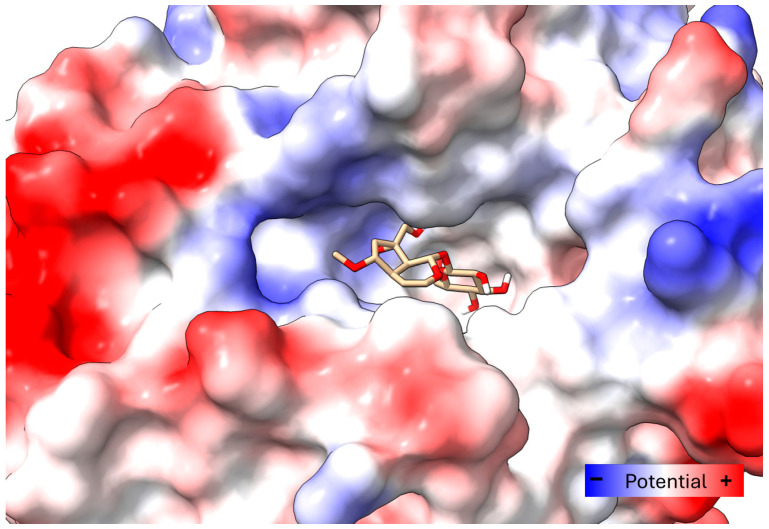
Coulombic electrostatic potential (ESP) maps for OYE and the 6*-O-*methylcatalpol ligand generated by Chimera X: the zones vary according to the color: red for negative potential, through white, to blue for positive potential. The bound ligand is shown in a stick model. The ligand’s position in the blue regions suggests favorable electrostatic interactions with negatively charged areas of the enzyme.

To better understand the ligand’s disposition, ChimeraX (Version 1.10.1) mapped the cavity’s surface. The hydrophobic zones and the electrostatic surface potential of the enzyme were identified. The models showed that the glycoside portion of the 6*-O-*methylcatalpol structure would have an affinity for zones with hydrophobic groups where the residues TYR200, PHE71, and TYR264 are located primarily (Figure 5). Comparing the surfaces with electrostatic potential reveals that the epoxide group is oriented toward residues such as ASN313 and ARG249, which generate positive polar surfaces (Figure 6).

## 3. Discussion

Countless reports document the trypanocidal activity of various natural products, including aqueous and alcoholic extracts, oleoresins, and essential oils derived from different plant sources [44]. However, there are no reports of trypanocidal activity against *T. cruzi* from extracts obtained from *Buddleja* species. Our results suggest that the methanolic extract of *B. globosa* exhibits trypanocidal activity, albeit to a lesser extent than the conventional antichagasic nifurtimox. MET’s trypanocidal activity could be at least partially linked to the presence of 6*-O-*methylcatapol, an iridoid related to catapol. Our results also suggest an effect associated with mitochondrial hyperpolarization. However, the extract’s effect may be enhanced by polyphenolic compounds, which could explain MET’s significant activity in contrast to the BG500 fraction, whose major iridoid component is 6*-O-*methylcatapol. The effect on *T. cruzi* may be related to the inhibition of OYE. This enzyme participates in maintaining the parasite’s intracellular redox balance. The observed ΔΨm hyperpolarization and antioxidant activity with MET could explain the selectivity of BG500 and MET, as these are protective factors in the host [38]; however, this link must be further demonstrated.

Catalpol, methylcatalpol, and several catalpol derivatives with aromatic substituents such as benzoyl, caffeoyl, cinnamoyl, and coumaroyl, linked at C6, have been isolated from various *Buddleja* species [45], specifically 6*-O-*methylcatalpol from *B. globosa* [39]. The 6*-O-*methylcatalpol characterized in this study was consistent with that reported by Hamedi et al. [40]. Similarly, the polyphenol content and antioxidant activity of *B. globosa* were consistent with previous reports [29]. However, MET’s antioxidant and biological activities and its fractions cannot be attributed solely to the presence of phenolic compounds; iridoids also contribute to these activities [33,46,47,48]. Thus, our findings may be linked to potential synergistic effects, as has already been reported [49,50,51,52], although combinatorial studies are required to validate this interaction.

Notably, two phenolic compounds in MET, quercetin and caffeic acid, have trypanocidal activity [23,53]. Quercetin may affect mitochondrial function in trypanosomatids by inhibiting enzymes, including heat shock proteins, topoisomerases, and kinases. Quercetin and caffeic acid also protect mitochondrial function by preventing increases in intracellular calcium levels [54]. The literature suggests that the polyphenols in *B. globosa* extract, such as rutin, can counteract the loss of ΔΨm induced by rotenone [55], and luteolin and chrysin have been linked to membrane collapse [55,56], suggesting that antagonistic interactions may occur in BG500 due to hyperpolarizing iridoids and polyphenols that cause membrane collapse.

An interesting finding was the broad selectivity of BG500 and MET, which is comparable to nifurtimox. It has been reported that catalpol’s hepatocyte ΔΨm hyperpolarizing effect is protective [38]. However, this may not be the case in parasites. T-cadinol, a sesquiterpene isolated from *Casearia sylvestris*, exhibits anti-*T. cruzi* activity but also causes ΔΨm hyperpolarization without Ca^2+^ mobility [57]. This effect was also observed with MET and the iridoid-enriched BG500 fraction in the present study. Whether this effect is linked to trypanocidal activity remains to be demonstrated because no direct molecular target was identified in the inner mitochondrial membrane [57]. However, docking analysis of several oxidoreductases involved in *T. cruzi* redox balance suggested that OYE may be a potential target.

Inhibiting the OYE enzyme may cause an imbalance in physiological processes and has been linked to changes in mitochondrial dynamics and shifts in ΔΨm [21,58]. Indeed, 6*-O-*methylcatalpol exhibited the lowest calculated affinity and, consequently, a higher probability of stable interaction with OYE and trypanothione reductase. Nevertheless, the expected binding affinity of 6*-O-*methylcatalpol is moderate and comparable to that of the naturally occurring substrate β-lapachone [58]. It is also lower than the binding affinity for the cofactor FMN, menadione [43,58], and benzimidazolequinone derivatives [59]. Nevertheless, the isoalloxazine ring of 6-O-methylcatalpol correlates with that of the simulated FMN, which is the biologically active part of the cofactor.

Our study suggests that there is a hydrophobic interaction between 6-*O*-methylcatalpol and OYE. While such interactions are common in molecular recognition, they tend to be less specific and weaker than other interactions, like hydrogen bonding or stronger π-π stacking, which is observed with menadione. Consequently, the affinity between 6-*O*-methylcatalpol and OYE is a significant limitation that may not adequately account for a strong pharmacological effect. Additionally, by not explicitly including FMN in our simulations, we simplified the model, which may have led to a decrease in the reported docking energy. This simplification could underestimate the actual affinity of the ligand in a more physiological context where the cofactor is present. Therefore, while 6-*O*-methylcatalpol shows potential as a hit for OYE, it requires further analysis to be validated as a lead compound. Further studies are essential because docking is a static tool that simulates only one ideal state, without accounting for the molecular dynamics or thermal fluctuations that occur in real biological systems. Predicting a single binding mode does not guarantee biological activity, as it often overlooks critical factors like the entropic cost of binding and desolvation effects. Moreover, the mechanistic hypothesis proposed herein is based on correlation rather than proven causality. To enhance the robustness of our findings, molecular dynamics studies or more advanced methods for estimating binding free energies, such as Generalized Born Surface Area or Poisson-Boltzmann Surface Area calculations, are necessary. These methods consider energy changes associated with ligand solvation and the entropic effects arising from the ligand’s interaction with the enzyme’s active site. Additionally, biological and biochemical validation studies comparing menadione’s inhibitory kinetic parameters (including Ki) with purified OYE, as well as site-directed mutagenesis experiments, are needed.

## 4. Materials and Methods

### 4.1. Plant Material

Leaves of *Buddleja globosa* were collected in Peralillo, commune of Peralillo, province of Colchagua, Region of Libertador General Bernardo O’Higgins, Chile (coordinates: −34.4731757; −71.4740390), in January 2024. The plant material was taxonomically identified by forest engineer Scarlett Denisse Norambuena. A voucher specimen was deposited in the SQF Herbarium of the University of Chile (voucher No. 22899) on 14 May 2024. *B. globosa* is not listed as a protected or endangered species in Chile. The material was collected from non-protected areas for non-commercial research purposes. In accordance with national regulations and institutional guidelines, no specific collection permit was required.

### 4.2. Extration Procedures and MET Fractionation

**Scheme 1:**
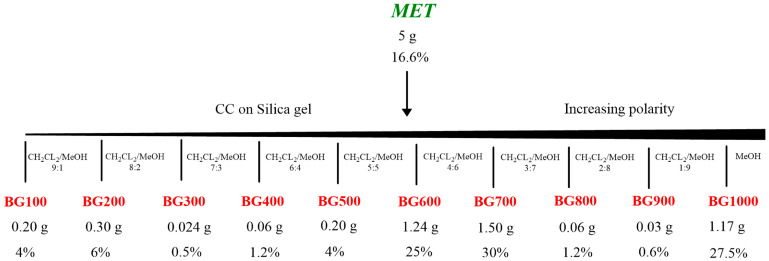
Scheme of fractionation of MET. CS100–1000: codes of the fractions obtained via column chromatography (CC) on silica gel; the absolute amount and yield percentages of the fractions are indicated. Increasing polarity: increasing polarity of solvent mixtures.

The crude methanol extract was obtained from 30 g of the dried aerial parts of *B. globosa* using ultrasound-assisted maceration with an Elmasonic S 10 H ELMA device (ELMA, Singen, Germany) for 30 min. This extraction was performed four times, and the combined extracts were dried using a rotary evaporator under reduced pressure at 50 °C. From 5 g of the dried methanolic extract, column chromatography on silica gel-60 (Merck KGaA, Darmstadt, Germany) was conducted. The chromatography device used a linear gradient of 0 to 100% dichloromethane to methanol, This process produced ten fractions (labeled as BG100–BG1000) (Scheme 1). After concentrating these fractions to dryness using 40–50 °C, depending on the solvent used, they were analyzed using thin-layer chromatography (TLC) on Merck GF-254 type 60 silica gel. The same eluent was used, and the spots were visualized with a vanillin-sulfuric acid reagent.

The iridoid 6*-O-*methylcatalpol was characterized through NMR analysis from fraction BG500.

### 4.3. Characterization via Nuclear Magnetic Resonance (NMR)

The ^1^H and ^13^C NMR spectra were recorded on a Varian 400 MHz spectrometer (running at 400 MHz for ^1^H and 100* *MHz for ^13^C). The spectra were obtained in CD_3_OD using tetramethylsilane (TMS) (Merck) as an internal standard. The chemical shifts (δ) are given in ppm and coupling constants in Hertz. The analyzed sample volume was 0.6 mL at a concentration of 41.666 µL/mL in deuterated methanol.

### 4.4. UPLC-ESI-MS/MS Analysis from MET

Ultra-performance liquid chromatography–electrospray tandem mass spectrometry coupled to tandem mass spectrometry (MS/MS) was performed following the methodology described elsewhere, with minor modification [60,61,62] in a 4500 triple quadrupole mass spectrometer controlled by Analyst^®^ 1.6.2 software (AB Sciex™, Darmstadt, Germany) and equipped with an ionization electrospray Turbo V attached to an Eksigent Ekspert Ultra 100 liquid chromatograph with an autosampler system (AB Sciex™, Concord, ON, Canada). The electrospray was employed in negative mode considering the following parameters: curtain gas = 30 psi, collision gas = 10 psi, ion spray voltage = −4500 V, temperature = 650 °C, ion source gas 1 = 50 psi, ion source gas 2 = 50 psi, and input potential = −10 V. The chromatographic separation was performed considering a gradient elution with two mobile phases: (A) 0.1% formic acid and (B) methanol. The gradient was carried out as follows: 0.1 min, 5% B; 1–12 min, 5–50% B; 12–13 min 50–50% B; 13–14 min, 50–5% B; and 14–15 min, 5% B. The injection volume was 10 μL with a flow rate of 0.5 mL/min and an end-capped column (LiChrospher 100 RP-18; 125 mm, 4 mm, 5 μm; Merck, Darmstadt, Germany) maintained at 40 °C. All analyses were performed in quadruplicate using a concentration sample of 8800 µg/mL. Quantification of compounds was performed using calibration curves constructed with commercial standards (Merck KGaA, Darmstadt, Germany). Peaks were selected based on their retention time consistency with authentic standards, expected MRM transitions, and overall signal quality. Only well-defined peaks with appropriate shape and reproducibility across replicates were considered. Peak integration was performed using Analyst^®^ software and manually reviewed when necessary.

Limits of detection (LOD), limit of quantification (LOQ), and the R2 of the plotted graphs were as follows: syringic acid (LOD = 55 ppb, LOQ = 167 ppb, and R2 = 0.9995); chlorogenic acid (LOD = 51 ppb, LOQ = 154 pbb, and R2 = 0.9994); caffeic acid (LOD = 142 ppb, LOQ = 430 ppb, and R2 = 0.9976); cryptochlorogenic acid (LOD = 0.05 ppb, LOQ = 0.17 ppb, and R2 = 0.9945); chrysin (LOD = 49 ppb, LOQ = 150 ppb, and R2 = 0.9997); rutin (LOD = 249 ppb, LOQ = 756 ppb, and R2 = 0.9939); quercetin (LOD = 67 ppb, LOQ = 203 ppb, and R2 = 0.9969); luteolin (LOD = 136 ppb, LOQ = 412 ppb, and R2 = 0.9965).

### 4.5. Determination of Total Phenolic Content (TPC) Assay

The total phenolic content of the different crude extracts was assessed spectrophotometrically using the Folin–Ciocalteu assay [63]. Five hundred microliters of the freshly prepared 10X diluted Folin–Ciocalteu reagent (125 µL) (Merck KGaA, Darmstadt, Germany) was mixed with an aliquot of 25 μL of the MET extract or the fractions at different concentrations (800, 500, and 600 µg/mL) and left to stand for 5 min at room temperature. To neutralize the mixture, 100 μL of sodium bicarbonate solution (Merck KGaA, Darmstadt, Germany) (7.5%, *w*/*v*) was added, and the resulting mixture was kept in the dark at room temperature for 60 min. Following incubation, the absorbance of the solution was measured at 765 nm using a UV-vis spectrophotometer (Allsheng AMR-100). Gallic acid (Sigma Aldrich, St. Louis, MO, USA) (0–180 mg/L) was used as a reference standard, and the respective standard curve was constructed.

### 4.6. Determination of Total Flavonoid Content (TFC)

The total flavonoid content of the extracts was determined using the aluminum chloride colorimetric method according to the procedure adapted from Ref. [64]. Briefly, 30 μL of plant extract at different concentrations (3000, 2400, and 2000 µg/mL) was further diluted with 250 μL of pure water and then mixed with 10 μL of aluminum chloride 10% (Merck KGaA, Darmstadt, Germany) and 10 μL of 1M potassium acetate (Merck KGaA, Darmstadt, Germany). The mixture was kept for 30 min at room temperature before recording the absorbance at 415 nm using a UV-vis spectrophotometer (Allsheng AMR-100). Quercetin (Sigma Aldrich, St. Louis, MO, USA) (0–180 mg/L) was used to construct the standard curve.

### 4.7. Antioxidant Capacity Assay

#### 4.7.1. Ferric Reducing Antioxidant Power (FRAP)

The extract’s FRAP was determined as previously described by Velázquez et al., with minor modifications [65]. The working FRAP solution was prepared daily by mixing 10 volumes of acetate buffer (pH 3.6, 0.3 M), 1 volume of 10 mM TPTZ ((2,4,6-tri(2-pyridyl)-s-triazine, Merck KGaA, Darmstadt, Germany), and 1 volume of 20 mM ferric chloride. Aliquots of FRAP solution (270 μL) were mixed with 30 μL of the MET or fractions at 1200, 600, and 300 µg/mL concentrations. The reaction mixtures were incubated for 30 min at 37 °C, after which the absorbance was measured at 594 nm using an Allsheng AMR-100 spectrophotometer with a plate reader. Trolox (50–350 μM, Sigma-Aldrich, St. Louis, MO, USA) was used to create the standard curve. The results are expressed as μmol Trolox equivalents per gram of dried plant (μmol TE/g).

#### 4.7.2. 2,2′-Azino-Bis(3-Ethylbenzothiazoline-6-Sulfonic Acid Radical Cation (ABTS^•+^)) Scavenging Activity

The activity of ABTS^•+^ was measured using the method described in [66], with modifications. In this assay, the ABTS radical, potassium persulfate oxidation, was mixed with the MET or fractions at 600, 300, and 125 µg/mL concentrations. A stock solution of ABTS at 7.00 mM was prepared one day prior to analysis. The working solution was prepared by diluting the stock solution to an absorbance value of 0.70 ± 0.02 at a wavelength of 734 nm in a 3 mL quartz cuvette, using a Helios Gamma spectrophotometer (model, UVG 1702E; Helios, Thermo Fischer Scientific, Horsham, UK). Then, 270 µL of the ABTS working solution was added to each well of the microtiter plate along with 30 µL of the extract or fraction sample. Absorbance was determined at 415 nm for 6 min [67], using a Multiskan™ GO UV/Vis microplate spectrophotometer (Thermo Fisher Scientific, Waltham, MA, USA).

ABTS free radical scavenging activity was calculated using the equation below:

ABTS free radical scavenging activity (%) = [(Abs_control_ − Abs_sample_)/Abs_control_] × 100.

where Abs_control_ is the absorbance of the ABTS radical + methanol and Abs_sample_ is the absorbance of the ABTS radical + pure compounds solution or Trolox. The results are expressed as Trolox equivalents [66].

#### 4.7.3. DPPH Free Radical Scavenging Activity

Antioxidant activity was measured using the method presented in [66] with minor modifications. We used the DPPH (2,2-diphenyl-1-picrylhydrazyl) radical assay with MET or fractions at 1200, 600, and 300 µg/mL concentrations. Absorbance was determined at 517 nm using a Multiskan™ GO UV/Vis microplate spectrophotometer (Thermo Fisher Scientific, USA) after 60 min in the dark.

DPPH free radical scavenging activity was calculated using the equation below:

DPPH free radical scavenging activity (%) = [(Abs_control_ − Abs_sample_)/(Abs_control_)] × 100

where Abs_control_ was the absorbance of the DPPH radical + ethanol and Abs_sample_ is the absorbance of the DPPH radical + pure compounds solution or Trolox. The results are expressed as Trolox equivalents [66].

### 4.8. Parasites

*T. cruzi* trypomastigotes (Dm28c strain), the infective, non-replicative form of the parasite, were collected from the supernatant of previously infected Vero cells (Fibroblasts from green monkey’s kidney, ATCC CCL-81) cultured in RPMI 1640 (Sigma-Aldrich, St. Louis, MO, USA) supplemented with 10% FBS, 2 mg/mL glutamine (Sigma-Aldrich, St. Louis, MO, USA) (pH 7.6), 100 UI/mL sodium penicillin, and 100 μg/mL streptomycin (Biological Industries, Beit Haemek, Israel), in a 5% CO^2^ atmosphere at 37 °C [68]. An initial density of 1 × 10^6^ parasites/mL (determined by means of microscopic counting in a Neubauer chamber) was cultured with the different concentrations of the extracts at 37 °C in RPMI for 24 h to determine the trypanocide activity.

### 4.9. Cell Viability Assay

First, 6.0 × 10^3^ Cells/mL Vero cells or 1.0 × 10^6^ trypomastigotes were seeded in 96-well or 24-well microplates. The plates were then incubated in RPMI medium at 37 °C with 5% CO_2_ for 24 h. Varying concentrations of MET, BG500, or nifurtimox (a tripanocidal control drug) were added to the plates. The final concentration of DMSO was kept below 0.5% *v*/*v*. After incubation, the cultures were washed with PBS to remove unbound substances. Then, 5 mg/mL of MTT (Merck, Darmstadt, Germany) and 0.22 mg/mL of phenazine (Merck, Darmstadt, Germany) were added to each well. The plates were then incubated at 37 °C for four hours to allow the living cells to reduce the MTT, converting it into an insoluble, colored product known as formazan. This formazan was dissolved by adding 10% sodium dodecyl sulfate (SDS) in 10 mM HCl, and the plates were incubated overnight at 37 °C. The resulting solution’s absorbance was measured colorimetrically using a plate reader (Asys Expert Plus©, Eugendorf, Austria) at a wavelength of 570 nm [69]. Absorbance is directly proportional to the number of viable cells. The results were expressed as a percentage of viable cells relative to the control group, and the IC_50_ was calculated using non-linear regression analysis with the least-squares method in GraphPad Prism 10. The selectivity index (SI) was calculated based on the IC_50_ data according to:

$$\frac{\mathrm{IC}50\;\mathrm{VERO}}{\mathrm{IC}50\;\mathrm{Trypomastigotes}}$$

### 4.10. Assessment of Mitochondrial Membrane Potential (ΔΨm) and Intracellular Calcium (Ca^2+^) Levels

*T. cruzi* trypomastigotes were cultured at a concentration of 5 × 10^6^ parasites/mL in 24-well flat-bottom plates. After 2 h of incubation—either with or without drugs—these parasites were exposed to tetramethylrhodamine methyl ester (TMRM) at a concentration of 200 nM for 30 min (Thermo Fischer, Waltham, MA, USA). For a positive control, the parasites were treated with carbonyl cyanide m-chlorophenylhydrazone (CCCP) at 25 μM for 10 min, which causes depolarization of the inner mitochondrial membrane. After treatment, the parasites were washed with phosphate-buffered saline (PBS) and moved to black 96-well flat-bottom plates, where they were treated with various extracts or catalpol (Merck, Darmstadt, Germany). All experimental conditions were analyzed in triplicate to ensure accuracy using untreated parasites as a control. The fluorescence of the samples was measured using a Tecan Spark fluorescence plate reader (Tecan, Männedorf, Switzerland) with an excitation wavelength of 535 nm and an emission wavelength of 595 nm [26]. The data were then quantified using GraphPad Prism 10, with the results expressed as the mean ± standard deviation (SD) from three independent experiments. For statistical analysis, we used a two-way ANOVA followed by Dunnett’s multiple comparisons test. A *p*-value of less than 0.05 was considered significant to indicate differences in TMRM signals between drug-treated and the non-treated control parasites.

Calcium levels were assessed as described by Conserva et al. [25]. Briefly, trypomastigotes (2 × 10^6^ cells/well) were pretreated with 5 μM of Fluo-4 AM (Molecular Probes, Invitrogen, Carlsbad, CA, USA) in PBS 1X, for 60 min at 37 °C in the dark. Then, the parasites were washed and treated with MET (500 μg/mL), BG500 (500 μg/mL), or catalpol (72.4 µg/mL). Fluorescence measurements were performed for 4 h using a Tecan Spark fluorescence plate reader (Tecan, Mannedorf, Switzerland), with the excitation and emission wavelengths of 485 and 535 nm, respectively. Maximum calcium levels were obtained using 0.5% Triton X-100, and the untreated parasites were used as a negative control. Statistical analysis was performed using analyses of variance (ANOVA) followed by Dunnett’s post hoc tests from three independent experiments.

### 4.11. Molecular Docking Study of 6-O-Methylcatalpol and Catalpol Binding with Old Yellow Enzyme (OYE)

The protein structure files for trypanothione reductase (TR), Old Yellow enzyme (OYE), cruzipain (Cp), and mitochondrial superoxide dismutase (TcSOD), enzymes involved in *T. cruzi* oxidoreductase metabolism, were sourced from the Protein Data Bank (PDB IDs: 1NDA, 4E2B, 1eWL, and 4dvh, respectively). The three-dimensional (3D) structure of 6*-O-*methylcatalpol was retrieved from the PubChem database, and the corresponding SMILES code was also obtained (Figure S12).

Several steps were taken to prepare the 3D structures for the ligand and the proteins. For the ligand, we added explicit hydrogen atoms, calculated and assigned partial atomic charges, and optimized its 3D geometry to ensure an energetically favorable conformation for docking. Similarly, we added the missing hydrogen atoms, assigned atomic charges, and refined their conformations for the proteins to ensure that they were in an accurate state for interaction with the ligand.

For molecular docking of the ligands with the proteins, we utilized SwissDock 2.0 [70,71] and Autodock Vina 1.2 [72,73] using the method of searching for attractive cavities [74,75] and energies of association (kcal/mol). SwissDock’s scoring function includes factors such as van der Waals forces, hydrogen bonds, hydrophobic interactions, and penalties for conformational entropy. In contrast, Vina computes interactions through a trilinear interpolation of precalculated grid maps and applies postprocessing minimization to the docked poses.

Vina’s sampling exhaustivity can range from 1 to 64, with a default value of 4; we used the maximum exhaustivity. To define the active site cavity, we utilized a grid box with dimensions of 20 × 20 × 20 and centered at the coordinates (OYE: 23, −5, −1; Cp: 0, 13, 6; TcSOD: 17, −11, 4; TR: 45, 57, 42). After docking, we performed a 2D and 3D visual analysis using Chimera X [76] to observe the main interactions between the ligands and the protein [77] (Figure S13). We conducted a redocking process, using the Maestro package, Schrödinger Release 2025-3 (Schrödinger, New York, NY, USA), to validate our in silico model and calculated the root mean square deviation (RMSD) between the initial and redocked poses.

### 4.12. Statistical Analyses

Statistical analyses were performed using Prism 10 software (GraphPad, San Diego, CA, USA). The data shown in the graphs are expressed as the mean ± SD of at least three independent experiments. Data from the determination of antioxidant activity and the assessment of ΔΨm were analyzed via ANOVA followed by Tukey’s and Dunnett’s multiple comparison post hoc tests, respectively. A D’Agostino–Pearson normality test was applied for the statistical tools used. Values were considered statistically significant at *p* < 0.05; details can be found in Figures S6 and S10 and Tables S1–S12.

## 5. Conclusions

This study reports, for the first time, the trypanocidal activity of an alcoholic extract from *B. globosa* leaves against the infective form of *T. cruzi*. The iridoid 6-*O*-methylcatalpol was characterized, various polyphenolic compounds were identified and quantified, and the antioxidant capacity of the extract and its fractions was assessed. Further studies are required to clarify the relative contribution of individual phenolic compounds and their potential synergistic interactions with iridoids in mediating the observed antiparasitic effects. MET, the BG500 fraction, and catalpol exhibited a hyperpolarizing effect on the mitochondrial membrane potential (ΔΨm) of *T. cruzi*. In silico analysis indicates that the enzyme OYE may be a potential molecular target for iridoids such as catalpol and 6-*O*-methylcatalpol. Nevertheless, additional research is needed to elucidate the relationship between the ΔΨm hyperpolarization and trypanocidal activity and to validate OYE as a suitable target.

## Figures and Tables

**Table 1:** ^1^H and ^13^C NMR data of 6-*O*-methylcatalpol (DMSO-d6).

N°	δ C	δ H (*J* in Hz)
1	93.81	5.07 (d, *J* = 9.7 Hz)
3	140.58	6.36 (d, *J* = 5.9 Hz)
4	102.59	5.03 (m)
5	35.95	2.35 (m)
6	87.15	3.65 (m)
7	57.77	3.68 (brs)
8	65.01	-
9	41.83	2.54 (t, *J* = 8.1 Hz)
10	60.16	4.16 (d, *J* = 13.1 Hz); 3.82 (d, *J* = 13.1 Hz)
OCH_3_	56.62	3.50 (s)
1′	98.29	4.79 (d, *J* = 7.8 Hz)
2′	73.42	3.30 (m)
3′	77.23	3.30 (m)
4′	70.36	3.30 (m)
5′	76.29	3.45–3.40 (m)
6′	61.54	3.93 (d, *J* = 12.2 Hz); 3.66 (m)

**Table 2:** Total phenolic content (TPC), total flavonoid content (TFC), ferric reducing antioxidant power (FRAP), and radical scavenging capacity (ABTS and DPPH) of MET and its fractions.

Extract or Fractions	TPC ^‡^ μmol GAE/g dw	TFC ^‡^ μmol QE/g dw	FRAP ^‡^ μmol TE/g dw	ABTS^+ ‡^ μmol TE/g dw	DPPH ^‡^ μmol TE/g dw
MET	1614.7 ± 105.9 ^a^	271.1 ± 3.0 ^a^	1021.5 ± 33.6 ^a^	849.0 ± 33.9 ^a^	849.7 ± 27.4 ^a^
BG100	NA	NA	46.2 ± 1.1 ^b^	NA	NA
BG200	44.8 ± 5.3 ^b^	655.6 ± 4.6 ^b^	226.0 ± 1.4 ^c^	NA	NA
BG300	662.5 ± 9.5 ^c^	NE	558.0 ± 1.7 ^d^	505.2 ± 10.9 ^b^	259.5 ± 5.6 ^b^
BG400	1036.1 ± 14.5 ^d^	NE	903.0 ± 1.4 ^e^	968.2 ± 18.6 ^c^	431.0 ± 11.4 ^c^
BG500	648.9 ± 5.5 ^c^	131.8 ± 4.9 ^c,d^	537.7 ± 2.9 ^d^	381.3 ± 17.9 ^d^	312.7 ± 9.5 ^d^
BG600	1224.9 ± 43.6 ^e^	118.2 ± 5.1 ^c^	749.9 ± 12.4 ^f^	517.3 ± 14.3 ^b^	605.3 ± 16.2 ^d^
BG700	308.2 ± 13.1 ^f,g^	186.8 ± 5.1 ^e^	559.2 ± 18.9 ^d^	212.4 ± 11.6 ^e^	326.9 ± 7.3 ^e^
BG800	213.3 ± 8.9 ^f^	221.1 ± 5.0 ^f^	273.9 ± 11.3 ^g^	198.6 ± 10.4 ^e^	110.3 ± 1.5 ^f^
BG900	331.1 ± 38.4 ^g^	145.2 ± 3.7 ^d^	333.2 ± 11.8 ^h^	194.2 ± 11.6 ^e^	118.6 ± 2.9 ^f^
BG1000	NA	136.0 ± 4.2 ^d^	150.5 ± 5.3 ^i^	83.5 ± 5.6 ^f^	NA

‡ See the list of abbreviations for their meanings. NA: no activity; NE: not evaluated. The values are the mean ± standard deviation from three independent assays. A one-way ANOVA was performed separately for each parameter, followed by Tukey’s multiple comparisons test. Different letters indicate statistical differences within each column. (For statistical details, see Tables S1–S10).

**Table 3:** UPLC-MS/MS identification and quantification of phenolic compounds of MET and the BG500 fraction.

Compound	Rt	[M–H]^−^	MS/MS	MET	BG500 Fraction
	(min)			mg/100g	mg/100g
Syringic acid	4.30	197.0	181.9	0.81 ± 0.04	1.48 ± 0.13
Chlorogenic acid	3.85	353.1	191.0	0.22 ± 0.03	0.36 ± 0.03
Caffeic acid	3.85	178.9	135.0	3.27 ± 0.27	0.04 ± 0.02
Rutin	5.61	609.0	299.8	154.94 ± 7.24	nd
Chrysin	11.38	253.0	208.9	2.44 ± 1.01	6.00 ± 1.25
Quercetin	8.10	301.0	150.9	45.88 ± 3.07	43.66 ± 3.60
Luteolin	7.98	285.0	133.0	6.34 ± 0.50	54.65 ± 1.07
Cryptochlorogenic acid	3.60	353.0	173.0	1.80 ± 0.12	nd

Rt: retention time; [M–H]^−^: molecular ion performed in negative ion mode. nd: not detected. Values represent the mean ± SD from quadruplicates.

**Table 4:** Biological activity of MET and BG500 on *T. cruzi* trypomastigotes (Dm28c strain).

	IC_50_ for Trypomastigotes (μg/mL)		IC_50_ for Vero Cells (μg/mL)		SI
		R2		R2	
MET	280 ± 3.5	0.99	>5000	0.97	>20
BG500 fraction	358 ± 4.2	0.98	>5000	0.98	>15
Nifurtimox	5.0 ± 0.5	0.96	184 ± 1.3 ‡	0.99	>20

IC_50_ values, calculated from a non-linear fitting using the least-squares method of the effect on parasite viability, correspond to the mean ± standard deviation (SD) of three individual experiments. The dose–response curves and concentration ranges used are indicated in Supplementary Figures S6–S8. ‡ Values are taken from ref [26].

**Table 5:** Estimated binding energy for 6*-O-*methylcatalpol as an inhibitor of *T. cruzi* oxidoreductases.

Enzyme	Binding Energy of the Inhibitor
Old Yellow enzyme (PDBID: 4E2B)	−6.411 Kcal/mol
Trypanothione reductase (PDBID: 1NDA)	−6.018 Kcal/mol
Cruzipain (PDBID:1eWL)	−5.841 Kcal/mol
SOD-Tc (PDBID:4dvh)	−5.051 Kcal/mol

## Data Availability

Data are contained within the article and Supplementary Materials.

## Supplementary Materials

The following supporting information can be downloaded at https://www.mdpi.com/article/10.3390/plants14172749/s1. Figure S1: ^1^H NMR of the BG500 fraction from the MET; Figure S2: ^13^C NMR of the BG500 fraction from the MET; Figure S3: DEPT-135 of the BG500 fraction from the MET; Figure S4: Cosy of the BG500 fraction from the MET; Figure S5: Cosy of the BG500 fraction from the MET; Figure S6: Effect of *B. globosa* MET on *T. cruzi* (Dm28c strain) trypomastigotes.; Figure S7: Effect of the BG500 fraction on *T. cruzi* (Dm28c strain) trypomastigotes; Figure S8: Effect of nifurtimox on *T. cruzi* (Dm28c strain) trypomastigotes; Figure S9: Confidence intervals of the effect size estimation analysis (Concentration Factor) of the MET, BG500 fraction, and catalpol on the *T. cruzi* ΔΨm; Figure S10: Statistical graphs obtained after the two-way post-Dunnett ANOVA for the incorporation of TMRM: (a) residual, (b) homoscedasticity, and (c) QQ plots; Figure S11: In green interaction of 6*-O-*methylcatalpol with FMN (hydrophobic contact); Figure S12: OYE protein obtained through the PDB database ID: 4E2B, and the docking parameters GRID box display from SwissDock software online; Figure S13: Molecular docking of catalpol with the OYE enzyme; Table S1: One-way ANOVA analysis of data from the TPC assay as indicated in Table 2, assuming a normal (Gaussian) distribution; Table S2: Tukey’s multiple comparison test for the TPC assay as indicated in Table 2, assuming a normal (Gaussian) distribution; Table S3: One-way ANOVA analysis of data from the TFC assay as indicated in Table 2, assuming a normal (Gaussian) distribution; Table S4: Tukey’s multiple comparison test for the TFC assay as as indicated in Table 2, assuming a normal (Gaussian) distribution; Table S5: One-way ANOVA analysis of data from the FRAP assay as indicated in Table 2, assuming a normal (Gaussian) distribution; Table S6: Tukey’s multiple comparison test for the FRAP assay as indicated in Table 2, assuming a normal (Gaussian) distribution; Table S7: One-way ANOVA analysis of data from the ABTS+ assay as indicated in Table 2, assuming a normal (Gaussian) distribution; Table S8: Tukey’s multiple comparison test for the ABTS+ assay as indicated in Table 2, assuming a normal (Gaussian) distribution; Table S9: One-way ANOVA analysis of data from the DPPH assay as indicated in Table 2, assuming a normal (Gaussian) distribution; Table S10: Tukey’s multiple comparison test for the DPPH assay as indicated in Table 2, assuming a normal (Gaussian) distribution; Table S11: Effect size estimation analysis (Time Factor) of MET, BG500 fraction, and catalpol on the *T. cruzi* mitochondrial membrane potential. Two-way ANOVA analysis (Alpha = 0.05); Table S12: Effect size estimation analysis (Concentration Factor) of the MET, BG500 fraction, and catalpol on the *T. cruzi* mitochondrial membrane potential. Two-way ANOVA analysis (Alpha = 0.05); Table S13: AutoDock Vina results showing the predicted binding energies (kcal/mol) for various conformations (models) of FMN and 6*-O-*mehylcatalpol bound to OYE. The models represent different orientations of each ligand within the active site.

## Abbreviations

The following abbreviations are used in this manuscript:

MET	Methanolic extract of *B. globosa* leaves
CD	Chagas disease
BNZ	Benznidazole
NADPH	Reduced forms of nicotinamide adenine dinucleotide phosphate
^13^C NMR	Carbon-13 nuclear magnetic resonance
^1^H NMR	Proton nuclear magnetic resonance
UPLC-ESI- MS/MS	Ultra-performance liquid chromatography–electrospray ionization–tandem mass spectrometry
LOD	Limits of detection
LOQ	Limit of quantification
Ppb	Parts per billion
TPC	Total phenolic content
TFC	Total flavonoid content
FRAP	Ferric reducing antioxidant power
ABTS^+^	2,2′-azinobis-3-ethylbenzthiazolin-6-Sulfonic acid
DPPH	2,2-diphenyl-1-picrylhydrazyl
GAE	Gallic acid equivalents
QE	Quercetin equivalent
TE	Trolox equivalent
Abs	Absorbance
RPMI	Roswell Park Memorial Institute Medium
FBS	Fetal bovine serum
MTT	(3-[4,5-dimethylthiazol-2-yl]-2,5 diphenyl tetrazolium bromide)
IC_50_	Concentration required to decrease 50% cell viability.
SI	Selectivity Index
SDS	Sodium dodecyl sulfate
DMSO	Dimethysulfoxide
ΔΨm	Mitochondrial membrane potential
TMRM	Tetramethylrhodamine methyl ester
CCCP	Carbonyl cyanide m-chlorophenylhydrazone
PBS	Phosphate-buffered saline
ANOVA	Analyses of variance
3D	Three-dimensional
2D	Two-dimensional
SD	Standard deviation
COSY	Correlation spectroscopy
HMBC	Heteronuclear multiple bond correlation
DEPT	Distortionless enhancement by polarization transfer
NA	No activity
NE	No evaluated
NADH	Reduced forms of the coenzymes nicotinamide adenine dinucleotide
FMN	Flavin mononucleotide
CD_3_OD	Deuterated methanol
TMS	Tetramethylsilane
OYE	Old Yellow Enzyme
TYR	Tyrosine
THR	Threonine
TR	Trypanothione reductase
PRO	Proline
ALA	Alanine
ESP	Coulombic electrostatic potential
CC	Column chromatography
Cp	Cruzipain
RMSD	Root-Mean-Square Deviation

## References

[1] Wilke A.B.B., Farina P., Ajelli M., Canale A., Dantas-Torres F., Otranto D., Benelli G. (2025). Human migrations, anthropogenic changes, and insect-borne diseases in Latin America. Parasit. Vectors.

[2] PAHO (2018). Actualización de la Estimación de la Enfermedad de Chagas en Los Países Endémicos de Las Américas 2018.

[3] Lynn M.K., Rodriguez Aquino M.S., Buendia Rivas C.A., Landrove A.M., Portillo de Juarez A.M., Aguila Ceron R., Garcia Lopez D., Nolan M.S., El Salvador Congenital Chagas t. (2025). Prenatal Chagas disease screening in Latin America: The current policy landscape and potential utility of an expanded maternal-familial *Trypanosoma cruzi* testing framework. Lancet Reg. Health Am..

[4] Zhu Y.S., Sun Z.S., Zheng J.X., Zhang S.X., Yin J.X., Zhao H.Q., Shen H.M., Baneth G., Chen J.H., Kassegne K. (2024). Prevalence and attributable health burdens of vector-borne parasitic infectious diseases of poverty, 1990-2021: Findings from the Global Burden of Disease Study 2021. Infect. Dis. Poverty.

[5] Cruz C.V., Rabinovich A., Moscatelli G., Moroni S., Gonzalez N., Garcia-Bournissen F., Ballering G., Freilij H., Altcheh J. (2024). Adverse events associated with benznidazole treatment for Chagas disease in children and adults. Br. J. Clin. Pharmacol..

[6] Berenstein A.J., Falk N., Moscatelli G., Moroni S., Gonzalez N., Garcia-Bournissen F., Ballering G., Freilij H., Altcheh J. (2021). Adverse Events Associated with Nifurtimox Treatment for Chagas Disease in Children and Adults. Antimicrob. Agents Chemother..

[7] OPS (2018). Guía Para El Diagnóstico Y El Tratamiento de la Enfermedad de Chagas.

[8] Filardi L.S., Brener Z. (1987). Susceptibility and natural resistance of *Trypanosoma cruzi* strains to drugs used clinically in Chagas disease. Trans. R. Soc. Trop. Med. Hyg..

[9] Wilkinson S.R., Taylor M.C., Horn D., Kelly J.M., Cheeseman I. (2008). A mechanism for cross-resistance to nifurtimox and benznidazole in trypanosomes. Proc. Natl. Acad. Sci. USA.

[10] Morillo C.A., Waskin H., Sosa-Estani S., Del Carmen Bangher M., Cuneo C., Milesi R., Mallagray M., Apt W., Beloscar J., Gascon J. (2017). Benznidazole and Posaconazole in Eliminating Parasites in Asymptomatic *T. cruzi* Carriers: The STOP-CHAGAS Trial. J. Am. Coll. Cardiol..

[11] Torrico F., Gascon J., Ortiz L., Alonso-Vega C., Pinazo M.J., Schijman A., Almeida I.C., Alves F., Strub-Wourgaft N., Ribeiro I. (2018). Treatment of adult chronic indeterminate Chagas disease with benznidazole and three E1224 dosing regimens: A proof-of-concept, randomised, placebo-controlled trial. Lancet Infect. Dis..

[12] Pinazo M.J., Forsyth C., Losada I., Esteban E.T., Garcia-Rodriguez M., Villegas M.L., Molina I., Crespillo-Andujar C., Gallego M., Ballart C. (2024). Efficacy and safety of fexinidazole for treatment of chronic indeterminate Chagas disease (FEXI-12): A multicentre, randomised, double-blind, phase 2 trial. Lancet Infect. Dis..

[13] Padilla A.M., Wang W., Akama T., Carter D.S., Easom E., Freund Y., Halladay J.S., Liu Y., Hamer S.A., Hodo C.L. (2022). Discovery of an orally active benzoxaborole prodrug effective in the treatment of Chagas disease in non-human primates. Nat. Microbiol..

[14] Betu Kumeso V.K., Kalonji W.M., Rembry S., Valverde Mordt O., Ngolo Tete D., Pretre A., Delhomme S., Ilunga Wa Kyhi M., Camara M., Catusse J. (2023). Efficacy and safety of acoziborole in patients with human African trypanosomiasis caused by *Trypanosoma brucei gambiense: A* multicentre, open-label, single-arm, phase 2/3 trial. Lancet Infect. Dis..

[15] Pinazo M.J., Malchiodi E., Ioset J.R., Bivona A., Gollob K.J., Dutra W.O. (2024). Challenges and advancements in the development of vaccines and therapies against Chagas disease. Lancet Microbe.

[16] de Menezes R.P.B., de Assis E.B., de Sousa N.F., de Souza J.M.S., Rodrigues K.A.D., Scotti L., Tavares J.F., da Silva M.S., Scotti M.T. (2025). Exploring Lamiaceae diterpenoids as potential multitarget therapeutics for leishmaniasis and chagas disease. Mol. Divers..

[17] Bridi R., Lino von Poser G., Gomez M., Andia M.E., Oyarzun J.E., Nunez P., Vasquez Arias A.J., Espinosa-Bustos C. (2021). Hepatoprotective species from the Chilean medicinal flora: *Junellia spathulata* (Verbenaceae). J. Ethnopharmacol..

[18] Díaz-Forestier J., León-Lobos P., Marticorena A., Celis-Diez J.L., Giovannini P. (2019). Native Useful Plants of Chile: A Review and Use Patterns. Econ. Bot..

[19] Ministerio De Salud (2011). Decreto Supremo 3: Aprueba Reglamento Del Sistema Nacional de Control de Los Productos Farmacéuticos de Uso Humano.

[20] Irigoin F., Cibils L., Comini M.A., Wilkinson S.R., Flohe L., Radi R. (2008). Insights into the redox biology of *Trypanosoma cruzi*: Trypanothione metabolism and oxidant detoxification. Free Radic. Biol. Med..

[21] Díaz-Viraqué F., Chiribao M.L., Trochine A., González-Herrera F., Castillo C., Liempi A., Kemmerling U., Maya J.D., Robello C. (2018). Old Yellow Enzyme from *Trypanosoma cruzi* Exhibits In Vivo Prostaglandin F2α Synthase Activity and Has a Key Role in Parasite Infection and Drug Susceptibility. Front. Immunol..

[22] Machado-Silva A., Cerqueira P.G., Grazielle-Silva V., Gadelha F.R., Peloso Ede F., Teixeira S.M., Machado C.R. (2016). How *Trypanosoma cruzi* deals with oxidative stress: Antioxidant defence and DNA repair pathways. Mutat. Res. Rev. Mutat. Res..

[23] Larit F., Elokely K.M., Nael M.A., Benyahia S., Leon F., Cutler S.J., Ghoneim M.M. (2021). Proposed Mechanism for the Antitrypanosomal Activity of Quercetin and Myricetin Isolated from *Hypericum afrum* Lam.: Phytochemistry, In Vitro Testing and Modeling Studies. Molecules.

[24] Marin C., Ramirez-Macias I., Lopez-Cespedes A., Olmo F., Villegas N., Diaz J.G., Rosales M.J., Gutierrez-Sanchez R., Sanchez-Moreno M. (2011). In vitro and in vivo trypanocidal activity of flavonoids from *Delphinium staphisagria* against Chagas disease. J. Nat. Prod..

[25] Conserva G.A., Costa-Silva T.A., Quiros-Guerrero L.M., Marcourt L., Wolfender J.L., Queiroz E.F., Tempone A.G., Lago J.H.G. (2021). Kaempferol-3-O-alpha-(3,4-di-E-p-coumaroyl)-rhamnopyranoside from *Nectandra oppositifolia* releases Ca^2+^ from intracellular pools of *Trypanosoma cruzi* affecting the bioenergetics system. Chem. Biol. Interact..

[26] Quintero-Pertuz H., Veas-Albornoz R., Carrillo I., Gonzalez-Herrera F., Lapier M., Carbono-Delahoz E., Del Olmo E., Feliciano A.S., Kemmerling U., Olea-Azar C. (2022). Trypanocidal effect of alcoholic extract of *Castanedia santamartensis* (Asteraceae) leaves is based on altered mitochondrial function. Biomed. Pharmacother..

[27] Bombaca A.C.S., Dossow D.V., Barbosa J.M.C., Paz C., Burgos V., Menna-Barreto R.F.S. (2018). TrypanocidalActivity of Natural Sesquiterpenoids Involves Mitochondrial Dysfunction, ROS Production and Autophagic Phenotype in *Trypanosoma cruzi*. Molecules.

[28] Otero C., Klagges C., Morales B., Sotomayor P., Escobar J., Fuentes J.A., Moreno A.A., Llancalahuen F.M., Arratia-Perez R., Gordillo-Fuenzalida F. (2023). Anti-Inflammatory Chilean Endemic Plants. Pharmaceutics.

[29] Torres-Vega J., Gomez-Alonso S., Perez-Navarro J., Alarcon-Enos J., Pastene-Navarrete E. (2021). Polyphenolic Compounds Extracted and Purified from *Buddleja globosa* Hope (Buddlejaceae) Leaves Using Natural Deep Eutectic Solvents and Centrifugal Partition Chromatography. Molecules.

[30] Pastene-Navarrete E., Torres-Vega J., Máthé Á., Bandoni A. (2021). Buddleja globosa Hope. Medicinal and Aromatic Plants of South America Vol. 2: Argentina, Chile and Uruguay.

[31] Dinda B. (2019). Occurrence and Distribution of Iridoids. Pharmacology and Applications of Naturally Occurring Iridoids.

[32] Houghton P.J., Hikino H. (1989). Anti-hepatotoxic activity of extracts and constituents of *Buddleja* species. Planta Med..

[33] Houghton P.J. (2003). *Buddleja globosa*: A Medicinal plant of Chile, their chemistry, biological activity and traditional uses. BLACPMA.

[34] Gonçalves G.A., Eifler-Lima V.L., von Poser G.L. (2022). Revisiting nature: A review of iridoids as a potential antileishmanial class. Phytochem. Rev..

[35] Liu J., Liu S., Yu M., Li J., Xie Z., Gao B., Liu Y. (2023). Anti-inflammatory effect and mechanism of catalpol in various inflammatory diseases. Drug Dev. Res..

[36] Wu C., Shi Z., Ge Q., Xu H., Wu Z., Tong P., Jin H. (2024). Catalpol promotes articular cartilage repair by enhancing the recruitment of endogenous mesenchymal stem cells. J. Cell Mol. Med..

[37] Zhang M., Qiang Y. (2023). Catalpol ameliorates inflammation and oxidative stress via regulating Sirt1 and activating Nrf2/HO-1 signaling against acute kidney injury. Environ. Toxicol..

[38] Gao X., Xu J., Liu H. (2020). Protective effects of catalpol on mitochondria of hepatocytes in cholestatic liver injury. Mol. Med. Rep..

[39] Duff R.B., Bacon J.S., Mundie C.M., Farmer V.C., Russell J.D., Forrester A.R. (1965). Catalpol and Methylcatalpol: Naturally Occurring Glycosides in Plantago and *Buddleia* Species. Biochem. J..

[40] Hamedi A., Zengin G., Aktumsek A., Selamoglu Z., Pasdaran A. (2020). In vitro and in silico approach to determine neuroprotective properties of iridoid glycosides from aerial parts of *Scrophularia amplexicaulis* by investigating their cholinesterase inhibition and anti-oxidant activities (Scrophulariaceae). Biointerface Res. Appl. Chem..

[41] Khan S., Ullah H., Zhang L. (2019). Review: Bioactive constituents form *Buddleja* species. Pak. J. Pharm. Sci..

[42] Stevens J.F., Revel J.S., Maier C.S. (2018). Mitochondria-Centric Review of Polyphenol Bioactivity in Cancer Models. Antioxid. Redox Signal..

[43] Yamaguchi K., Okamoto N., Tokuoka K., Sugiyama S., Uchiyama N., Matsumura H., Inaka K., Urade Y., Inoue T. (2011). Structure of the inhibitor complex of old yellow enzyme from *Trypanosoma cruzi*. J. Synchrotron Radiat..

[44] Barbosa H., Thevenard F., Reimao J.Q., Tempone A.G., Honorio K.M., Lago J.H.G. (2023). The Potential of Secondary Metabolites from Plants as Drugs or Leads against *Trypanosoma*-An Update from 2012 to 2021. Curr. Top. Med. Chem..

[45] Thabet A.A., Ayoub I.M., Youssef F.S., Al-Sayed E., Efferth T., Singab A.N.B. (2022). Phytochemistry, structural diversity, biological activities and pharmacokinetics of iridoids isolated from various genera of the family Scrophulariaceae Juss. Phytomedicine Plus.

[46] Xu Z., Zhang L., Li X., Jiang Z., Sun L., Zhao G., Zhou G., Zhang H., Shang J., Wang T. (2015). Mitochondrial fusion/fission process involved in the improvement of catalpol on high glucose-induced hepatic mitochondrial dysfunction. Acta Biochim. Biophys. Sin..

[47] Kucharska A.Z., Sokol-Letowska A., Oszmianski J., Piorecki N., Fecka I. (2017). Iridoids, Phenolic Compounds and Antioxidant Activity of Edible Honeysuckle Berries (*Lonicera caerulea* var. *kamtschatica* Sevast.). Molecules.

[48] Tenuta M.C., Loizzo M.R., Tundis R., Dugay A., Bouzidi C., Marie A., Acquaviva R., Cappello A.R., Deguin B. (2023). Iridoid- and flavonoid-enriched fractions of *Cornus sanguinea* and *Cornus mas* exert antioxidant and anti-inflammatory effects and inhibit key enzymes in the treatment of metabolic disorders. Food Funct..

[49] Mitra S., Tareq A., Das R., Bin Emran T., Nainu F., Chakraborty A.J., Ahmad I., Tallei T.E., Idris A.M., Simal-Gandara J. (2023). Polyphenols: A first evidence in the synergism and bioactivities. Food Rev. Int..

[50] Mieres-Castro D., Theoduloz C., Sus N., Burgos-Edwards A., Schmeda-Hirschmann G., Frank J., Jimenez-Aspee F. (2022). Iridoids and polyphenols from chilean Gaultheria spp. berries decrease the glucose uptake in Caco-2 cells after simulated gastrointestinal digestion. Food Chem..

[51] Przybylska D., Kucharska A.Z., Piórecki N., Sozanski T. (2024). The Health-Promoting Quality Attributes, Polyphenols, Iridoids and Antioxidant Activity during the Development and Ripening of Cornelian Cherry (*Cornus mas* L.). Antioxidants.

[52] Kwak J.H., Kim H.J., Lee K.H., Kang S.C., Zee O.P. (2009). Antioxidative iridoid glycosides and phenolic compounds from *Veronica peregrina*. Arch. Pharm. Res..

[53] Schmidt T.J., Khalid S.A., Romanha A.J., Alves T.M.A., Biavatti M.W., Brun R., Da Costa F.B., de Castro S.L., Ferreira V.F., de Lacerda M.V.G. (2012). The Potential of Secondary Metabolites from Plants as Drugs or Leads Against Protozoan Neglected Diseases—Part II. Curr. Med. Chem..

[54] Kempuraj D., Madhappan B., Christodoulou S., Boucher W., Cao J., Papadopoulou N., Cetrulo C.L., Theoharides T.C. (2005). Flavonols inhibit proinflammatory mediator release, intracellular calcium ion levels and protein kinase C theta phosphorylation in human mast cells. Br. J. Pharmacol..

[55] Seydi E., Rahimpour Z., Salimi A., Pourahmad J. (2019). Selective toxicity of chrysin on mitochondria isolated from liver of a HCC rat model. Bioorg Med. Chem..

[56] Chen P., Zhang J.Y., Sha B.B., Ma Y.E., Hu T., Ma Y.C., Sun H., Shi J.X., Dong Z.M., Li P. (2017). Luteolin inhibits cell proliferation and induces cell apoptosis via down-regulation of mitochondrial membrane potential in esophageal carcinoma cells EC1 and KYSE450. Oncotarget.

[57] Dos Santos A.L., Amaral M., Hasegawa F.R., Lago J.H.G., Tempone A.G., Sartorelli P. (2021). (-)-T-Cadinol-a Sesquiterpene Isolated from *Casearia sylvestris* (Salicaceae)-Displayed *In Vitro* Activity and Causes Hyperpolarization of the Membrane Potential of *Trypanosoma cruzi*. Front. Pharmacol..

[58] Murakami M.T., Rodrigues N.C., Gava L.M., Honorato R.V., Canduri F., Barbosa L.R., Oliva G., Borges J.C. (2013). Structural studies of the *Trypanosoma cruzi* Old Yellow Enzyme: Insights into enzyme dynamics and specificity. Biophys. Chem..

[59] Lopez-Lira C., Tapia R.A., Herrera A., Lapier M., Maya J.D., Soto-Delgado J., Oliver A.G., Graham Lappin A., Uriarte E. (2021). New benzimidazolequinones as trypanosomicidal agents. Bioorg. Chem..

[60] Varas Condori M.A., Arias-Sante M.F., Bridi R., Rincon-Cervera M.A., Porras O., Reyes-Jara A., de Camargo A.C. (2025). Phenolics of Maqui Leaf Residues Exhibit Antioxidant Properties Against Ozone-Induced Oxidation in Fish Model Systems. Antioxidants.

[61] Toledo-Merma P.R., Arias-Sante M.F., Rincon-Cervera M.A., Porras O., Bridi R., Rhein S., Sanchez-Contreras M., Hernandez-Pino P., Tobar N., Puente-Diaz L. (2024). Phenolic Fractions from Walnut Milk Residue: Antioxidant Activity and Cytotoxic Potential. Plants.

[62] de Camargo A.C., Speisky H., Bridi R., Nunez Pizarro P., Larena A., Pinaffi-Langley A., Shahidi F., Schwember A.R. (2022). Chickpeas from a Chilean Region Affected by a Climate-Related Catastrophe: Effects of Water Stress on Grain Yield and Flavonoid Composition. Molecules.

[63] Perez M., Dominguez-Lopez I., Lamuela-Raventos R.M. (2023). The Chemistry Behind the Folin-Ciocalteu Method for the Estimation of (Poly)phenol Content in Food: Total Phenolic Intake in a Mediterranean Dietary Pattern. J. Agric. Food Chem..

[64] Chang C.C., Yang M.H., Wen H.M., Chern J.C. (2002). Estimation of total flavonoid content in propolis by two complementary colorimetric methods. J. Food Drug Anal..

[65] Velásquez P., Rodríguez K., Retamal M., Giordano A., Valenzuela L.M., Montenegro G. (2017). Relation between composition, antioxidant and antibacterial activities and botanical origin of multi-floral bee pollen. J. Appl. Bot. Food Qual..

[66] De Camargo A.C., Vieira T., Regitano-D’Arce M.A.B., Calori-Domingues M.A., Canniatti-Brazaca S.G. (2012). Gamma radiation effects on peanut skin antioxidants. Int. J. Mol. Sci..

[67] Prior R.L., Wu X., Schaich K. (2005). Standardized methods for the determination of antioxidant capacity and phenolics in foods and dietary supplements. J. Agric. Food Chem..

[68] Pires S.F., DaRocha W.D., Freitas J.M., Oliveira L.A., Kitten G.T., Machado C.R., Pena S.D., Chiari E., Macedo A.M., Teixeira S.M. (2008). Cell culture and animal infection with distinct *Trypanosoma cruzi* strains expressing red and green fluorescent proteins. Int. J. Parasitol..

[69] Mosmann T. (1983). Rapid colorimetric assay for cellular growth and survival: Application to proliferation and cytotoxicity assays. J. Immunol. Methods.

[70] Bugnon M., Rohrig U.F., Goullieux M., Perez M.A.S., Daina A., Michielin O., Zoete V. (2024). SwissDock 2024: Major enhancements for small-molecule docking with Attracting Cavities and AutoDock Vina. Nucleic Acids Res..

[71] Grosdidier A., Zoete V., Michielin O. (2011). SwissDock, a protein-small molecule docking web service based on EADock DSS. Nucleic Acids Res..

[72] Eberhardt J., Santos-Martins D., Tillack A.F., Forli S. (2021). AutoDock Vina 1.2.0: New Docking Methods, Expanded Force Field, and Python Bindings. J. Chem. Inf. Model..

[73] Trott O., Olson A.J. (2010). Software News and Update AutoDock Vina: Improving the Speed and Accuracy of Docking with a New Scoring Function, Efficient Optimization, and Multithreading. J. Comput. Chem..

[74] Röhrig U.F., Goullieux M., Bugnon M., Zoete V. (2023). Attracting Cavities 2.0: Improving the Flexibility and Robustness for Small-Molecule Docking. J. Chem. Inf. Model..

[75] Zoete V., Schuepbach T., Bovigny C., Chaskar P., Daina A., Röhrig U.F., Michielin O. (2016). Attracting Cavities for Docking. Replacing the Rough Energy Landscape of the Protein by a Smooth Attracting Landscape. J. Comput. Chem..

[76] Pettersen E.F., Goddard T.D., Huang C.R.C., Meng E.E.C., Couch G.S., Croll T.I., Morris J.H., Ferrin T.E. (2021). UCSF ChimeraX: Structure visualization for researchers, educators, and developers. Protein Sci..

[77] Goddard T.D., Huang C.C., Meng E.C., Pettersen E.F., Couch G.S., Morris J.H., Ferrin T.E. (2018). UCSF ChimeraX: Meeting modern challenges in visualization and analysis. Protein Sci..

