# Supplementary material for: Buddleja globosa Leaf Methanolic Extract Acts Against Trypanosoma cruzi Parasites by Inducing Mitochondrial Inner Membrane Hyperpolarization
Source: Plants (Basel). 2025 Sep 2;14(17):2749. doi: 10.3390/plants14172749 (PMC12430229; doi:10.3390/plants14172749)
Supplement: Supplementary file 1 [file plants-14-02749-s001.zip › plants-3720573-supplementary.pdf]

plants

MDPI

Buddleja globosa Leaf Methanolic Extract Acts Against *Trypanosoma cruzi* Parasites by Inducing Mitochondrial Inner Membrane Hyperpolarization

Helena Quintero-Pertuz [1,2], Vicente Valenzuela-Bass [1], Michel Lapier [3], José Ortega-Campos [4,5], Sebastián Alfaro [5], Gilsane Lino von Poser [6], Christian Espinosa-Bustos [7], Adriano Costa de Camargo [8], Fabiola González-Herrera [9], Juan D. Maya [5,*] and Raquel Bridi [1,*]

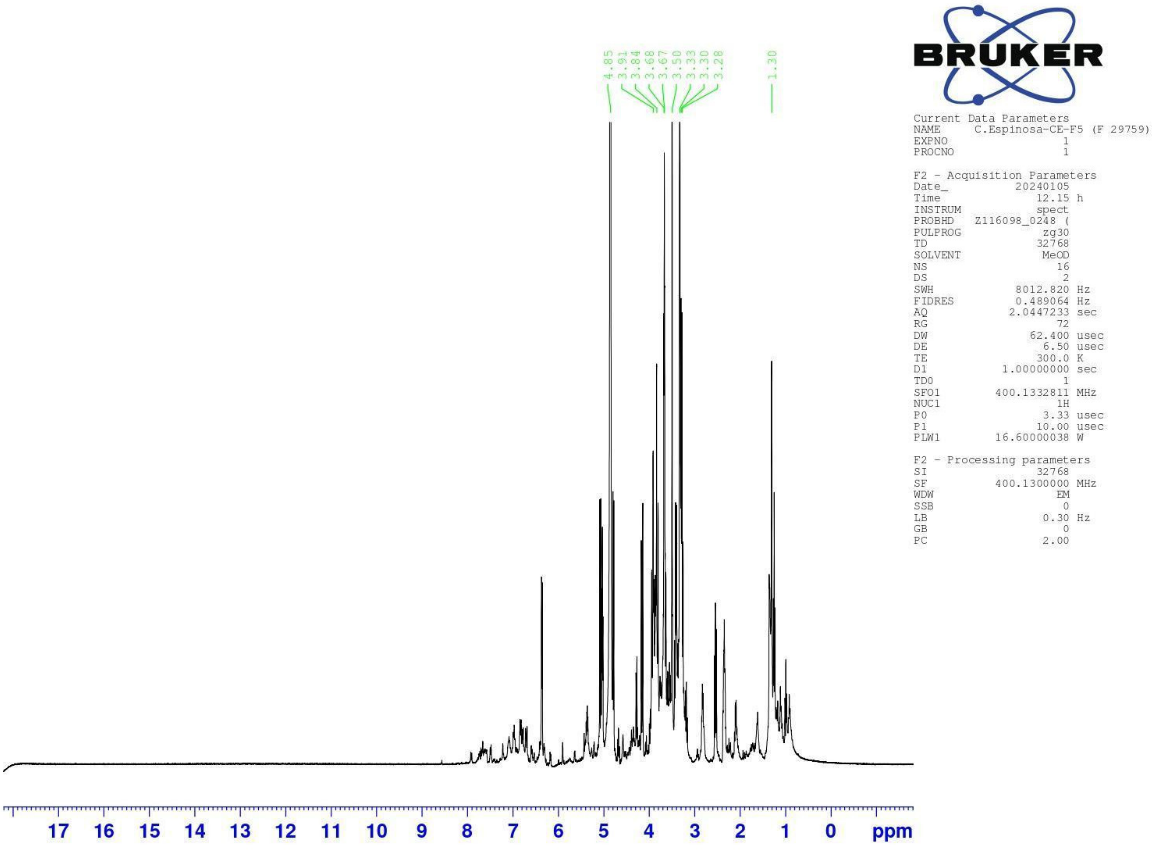

Figure S1. 1H NMR of BG500 fraction from the MET.

 https://doi.org/10.3390/plants14172749

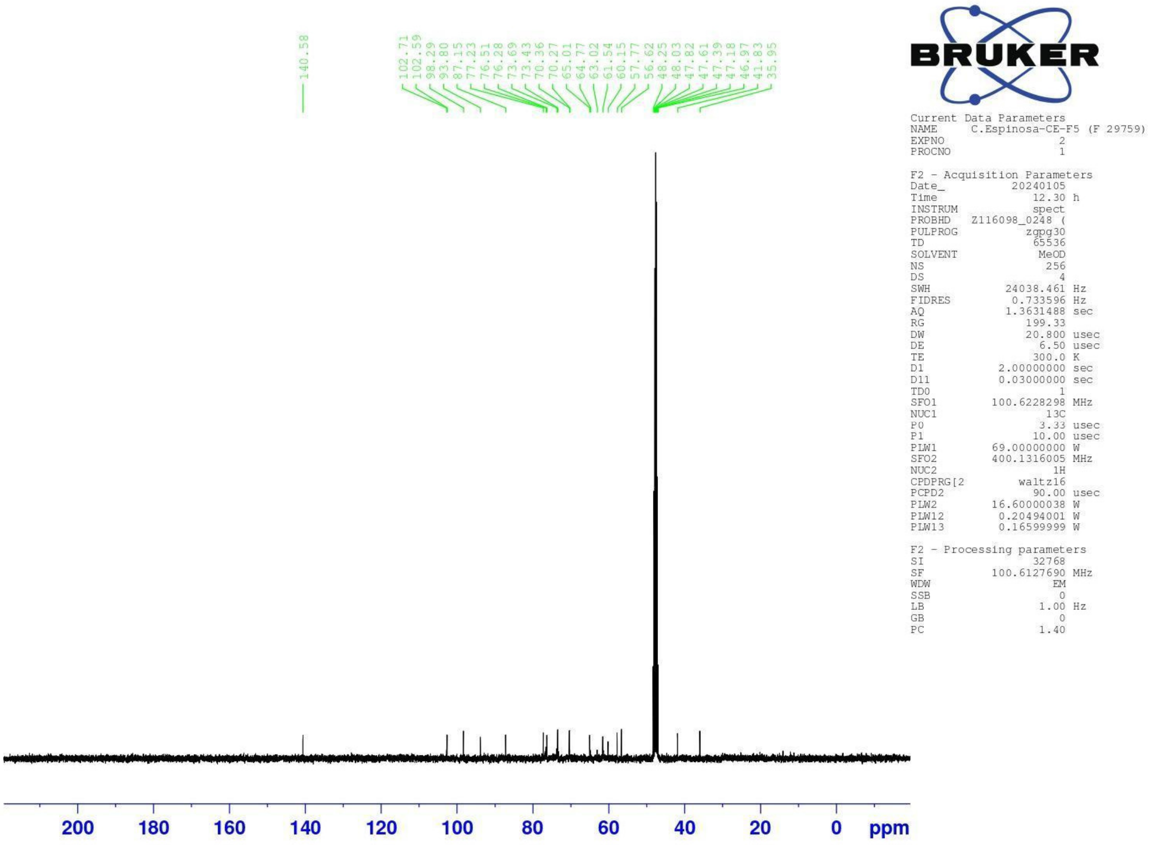

Figure S2. ^{13}C NMR of BG500 fraction from the MET.

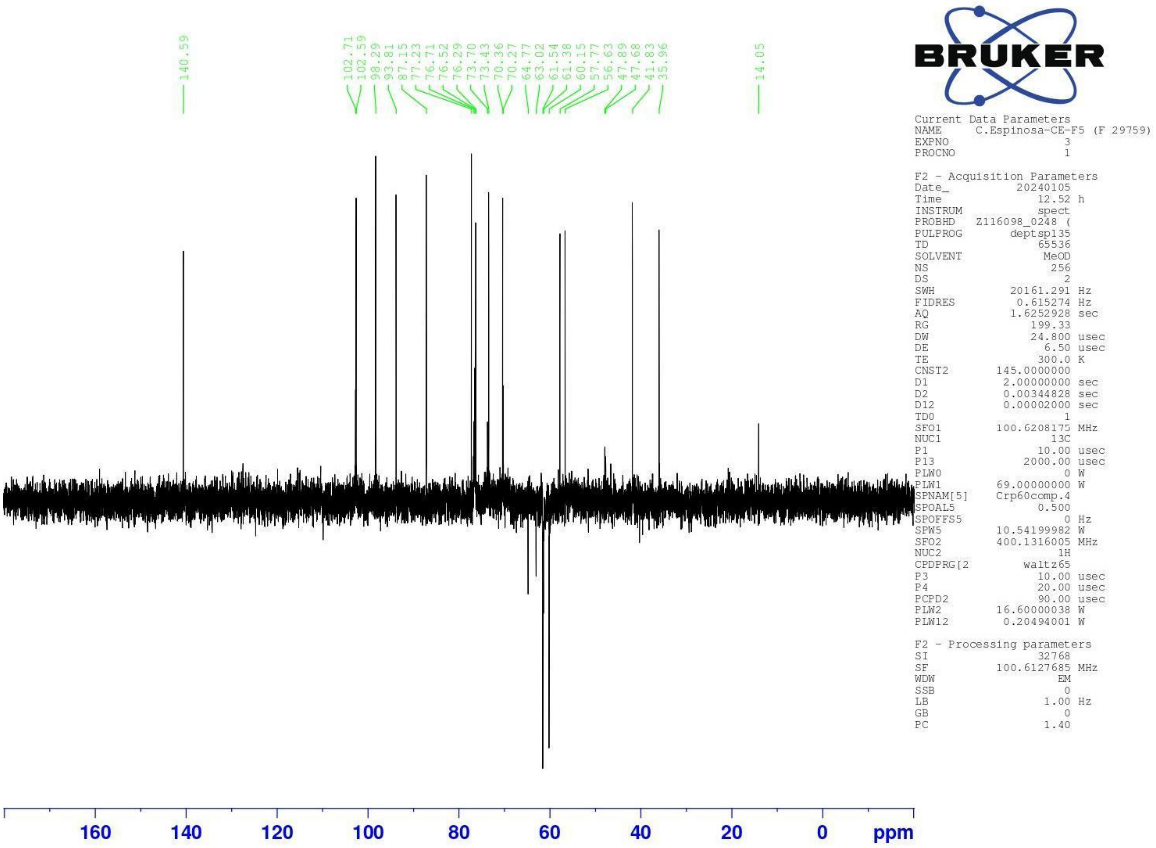

Figure S3. DEPT-135 of BG500 fraction from the MET.

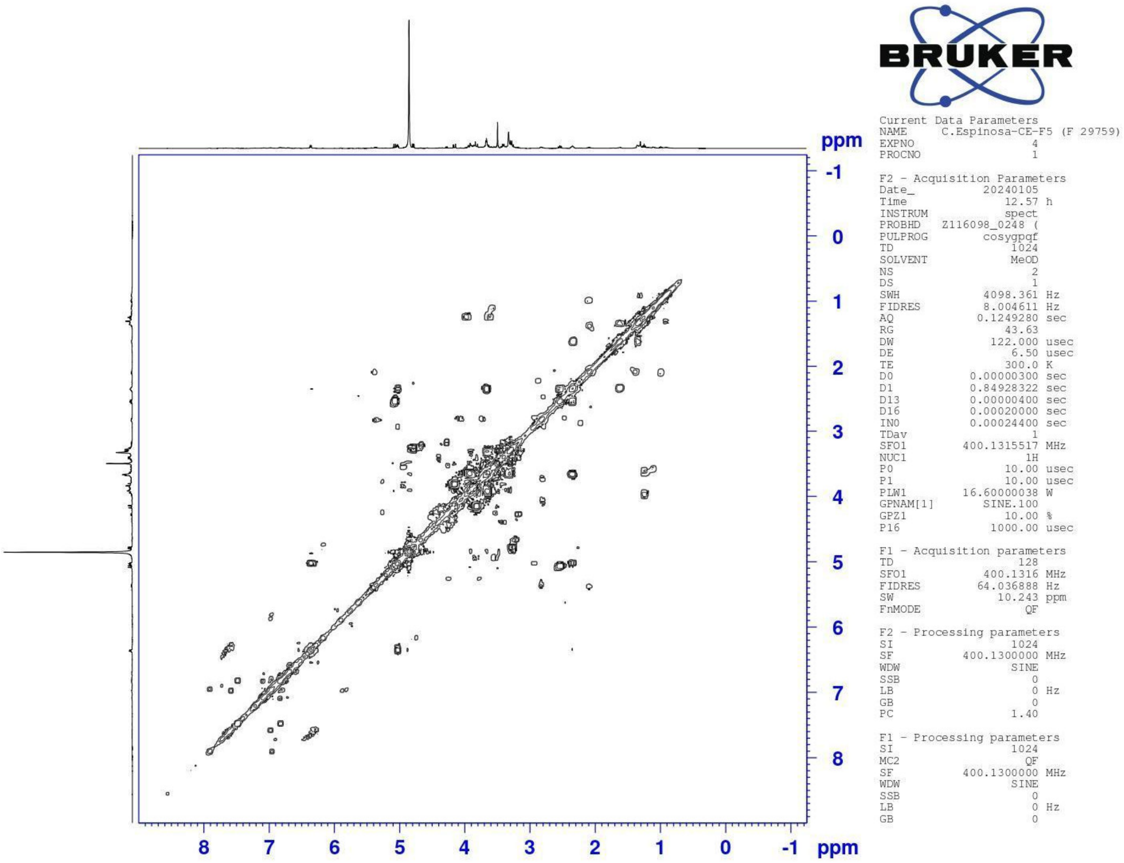

Figure S4. Cosy of BG500 fraction from the MET.

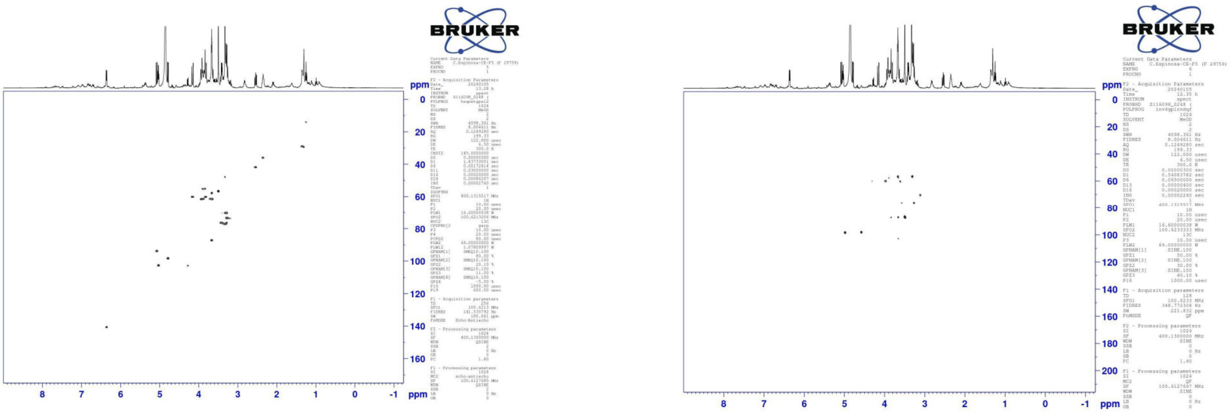

Figure S5. Cosy of BG500 fraction from the MET.

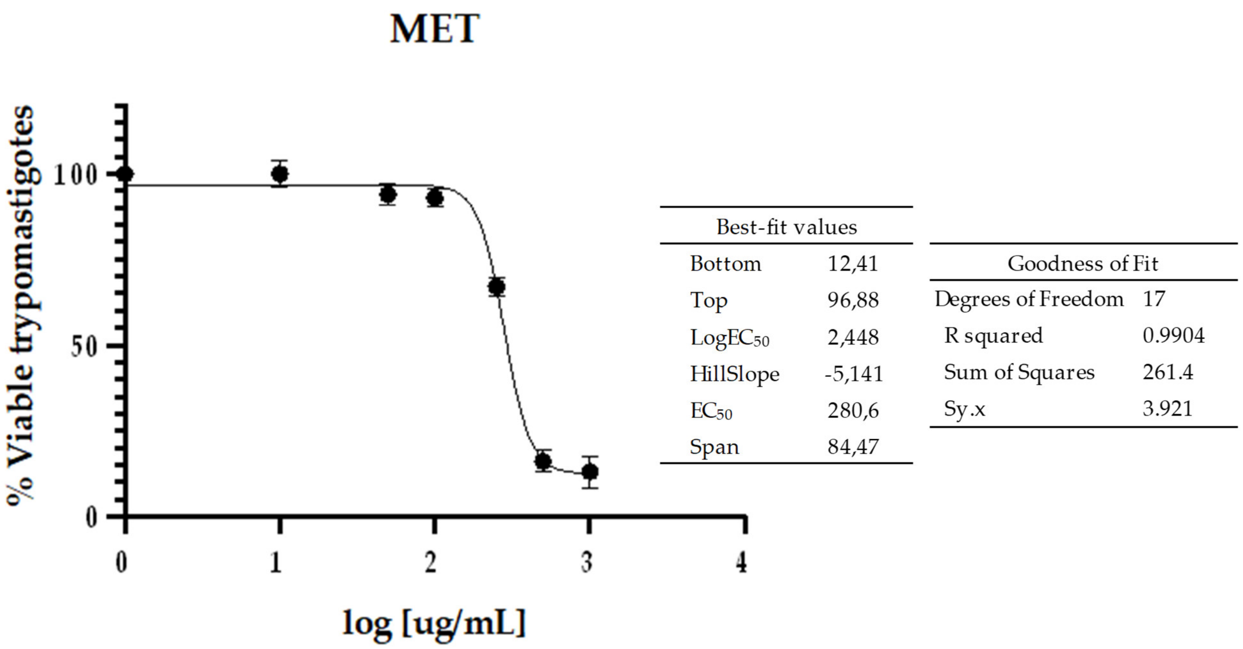

Figure S6. Effect of *B. globosa* MET on *T. cruzi* (Dm28c strain) trypomastigotes. 1.0 × 10^6 trypomastigotes were incubated in RPMI at 37 °C and 5% CO_2 for 24 h with 0; 10; 50; 100; 250; 500 and 1000 µg/mL. Viability was assessed by using the MTT colorimetric assay and expressed as a percentage of viable cells relative to the control, untreated parasites. IC_{50} was calculated using a non-linear regression analysis with the least-squares method in GraphPad Prism 10.

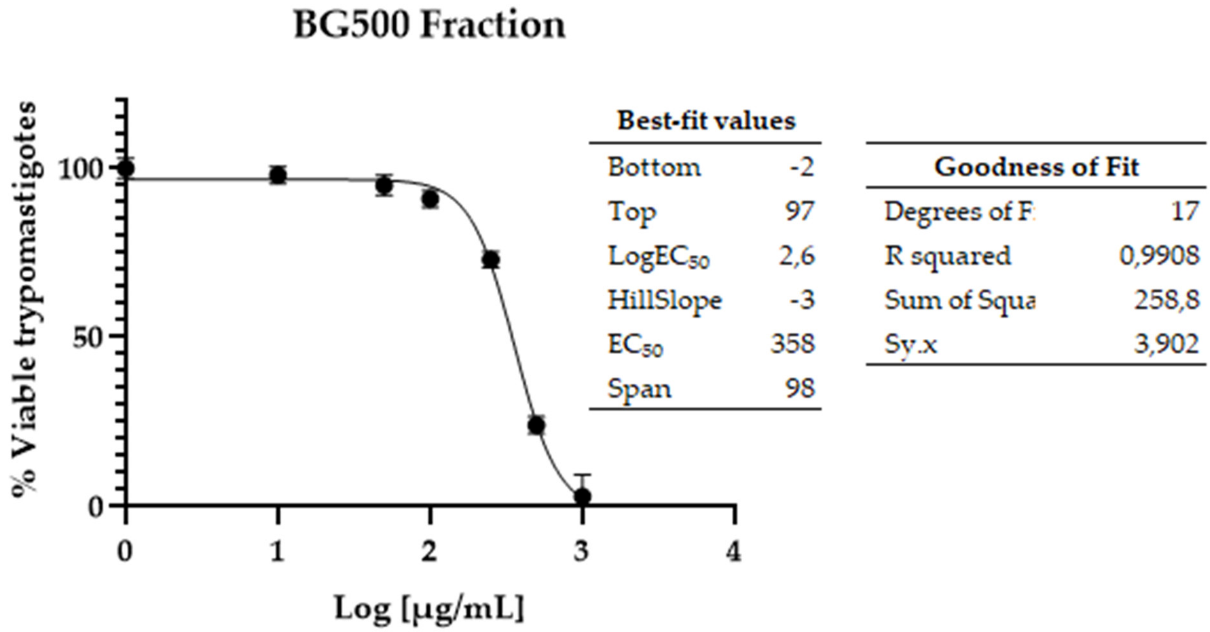

Figure S7. Effect of *B. globosa* BG500 fraction on *T. cruzi* (Dm28c strain) trypomastigotes. 1.0 × 10^6 trypomastigotes were incubated in RPMI at 37 °C and 5% CO_2 for 24 h with 0; 10; 50; 100; 250; 500 and 1000 µg/mL. Viability was assessed by using the MTT colorimetric assay and expressed as a percentage of viable cells relative to the control, untreated parasites. IC_{50} was calculated using a non-linear regression analysis with the least-squares method in GraphPad Prism 10.

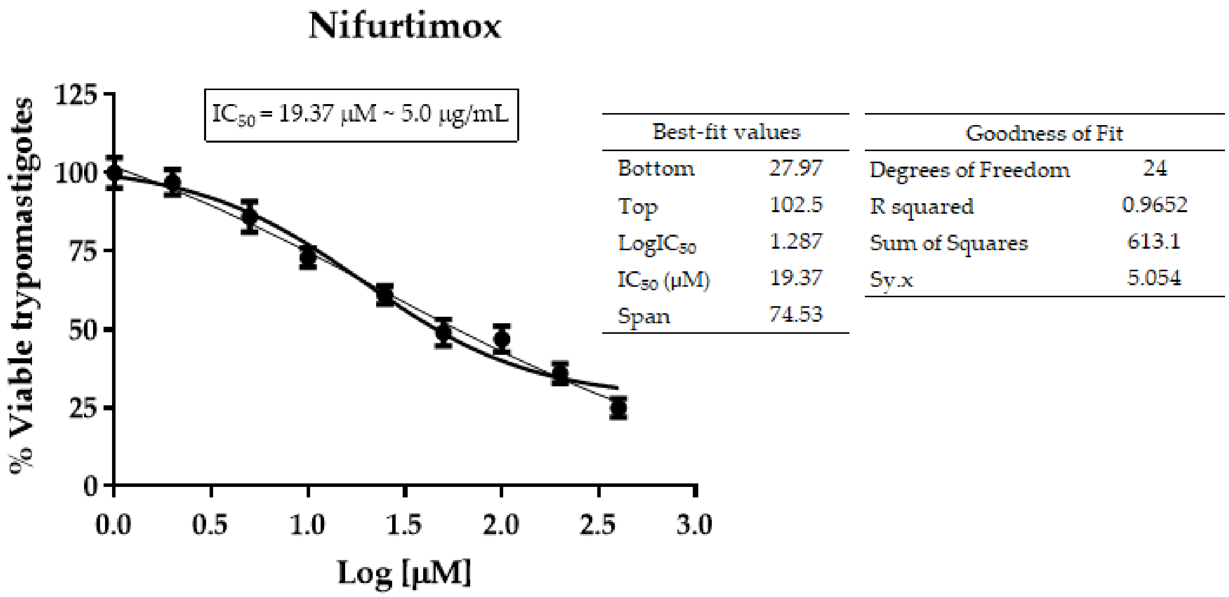

Best-fit values	
Bottom	27.97
Top	102.5
LogIC_{50}	1.287
IC_{50} (µM)	19.37
Span	74.53

Goodness of Fit	
Degrees of Freedom	24
R squared	0.9652
Sum of Squares	613.1
Sy.x	5.054

Figure S8. Effect of nifurtimox on *T. cruzi* (Dm28c strain) trypomastigotes. 1.0×10^6 trypomastigotes were incubated in RPMI at 37 °C and 5% CO_2 for 24 h with 0; 10; 50; 100; 250; 500 and 1000 µg/mL MET. Viability was assessed by using the MTT colorimetric assay and expressed as a percentage of viable cells relative to the control, untreated parasites. IC_{50} was calculated using a non-linear regression analysis with the least-squares method in GraphPad Prism 10.

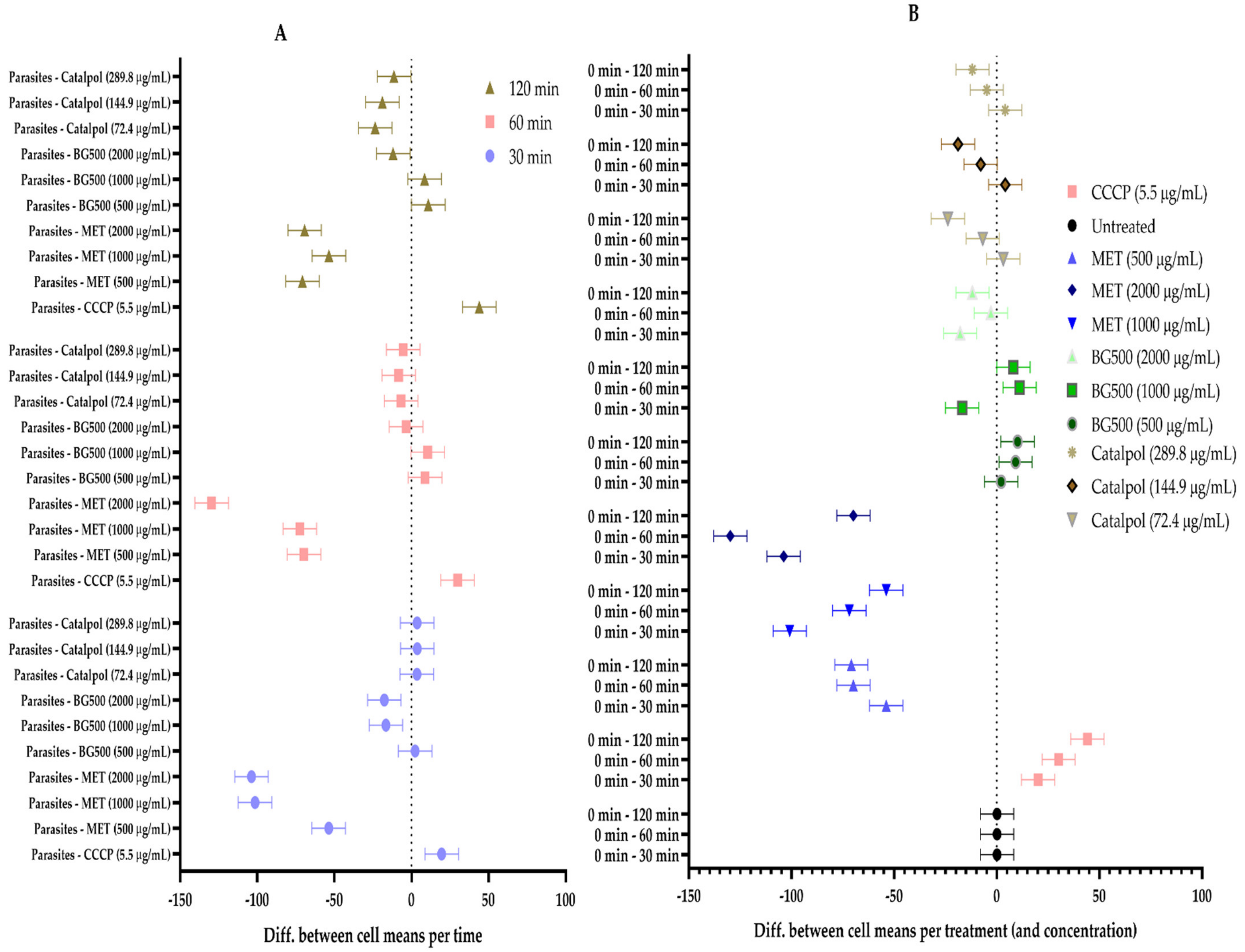

Figure S9. Confidence intervals of the effect size estimation analysis (Concentration Factor) of the MET, BG500 fraction and catalpol on the *T. cruzi* ΔΨm. Two-way ANOVA analysis Dunnett's multiple comparison test test.

(a) (b) (c)

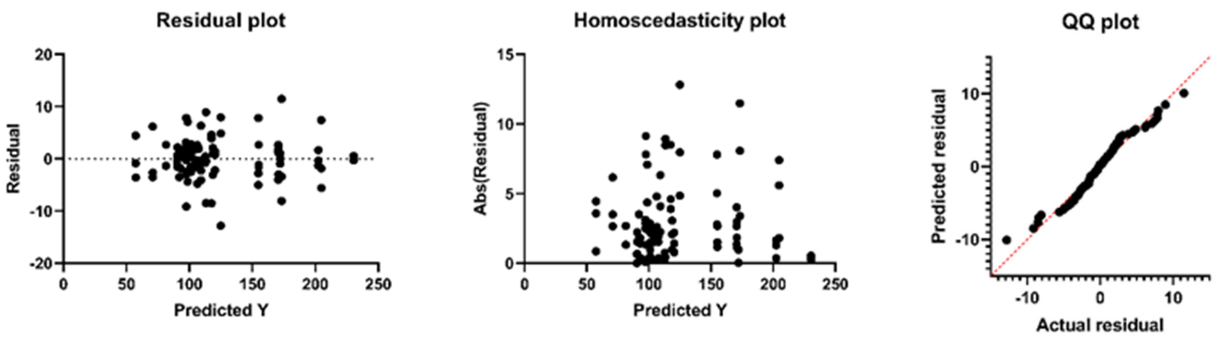

Figure S10. Statistical graphs obtained after the two-way post-Dunnett ANOVA for the incorporation of TMRM: **(a)** residual, **(b)** homoscedasticity and **(c)** QQ plots.

A)

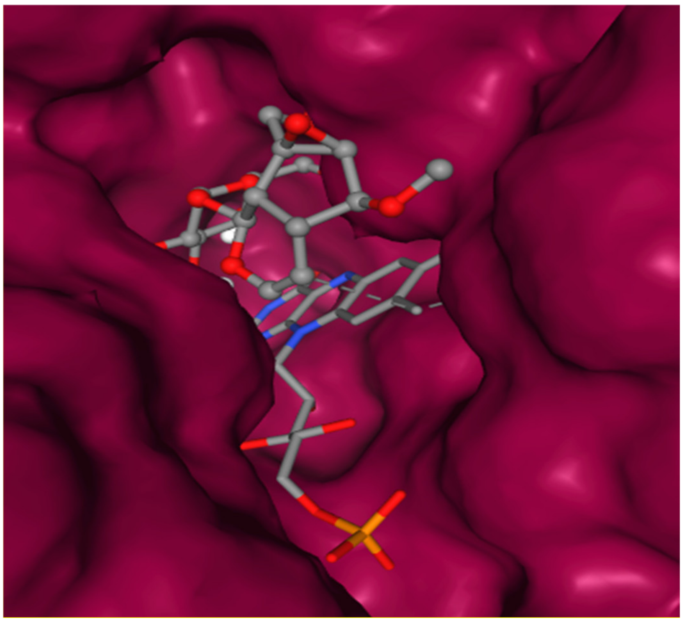

B)

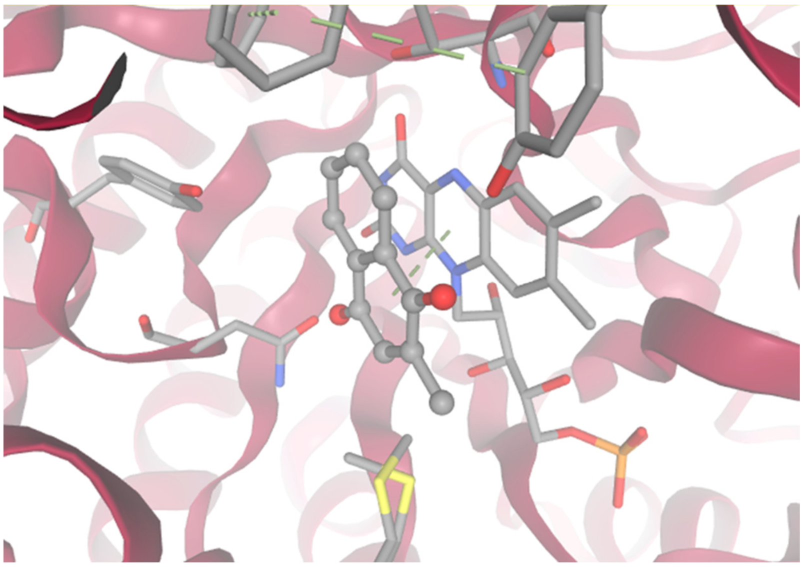

Figure S11. This scheme compares the molecular interactions between **flavin mononucleotide (FMN)** and two different ligands. In **A**, the interaction of **6-*O*-methylcatalpol** (in green) with FMN is shown, where the primary contact is **hydrophobic**. In **B**, the interaction of the classical inhibitor **menadione** with FMN is illustrated, which can form π–π stacking interactions, typically indicating a stronger and more specific binding than the one observed in A. .

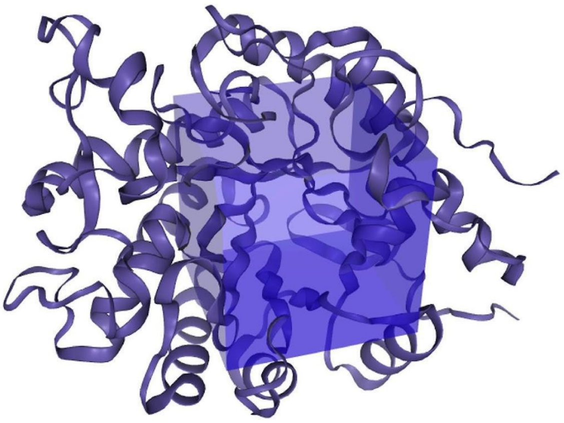

SMILES Ligand 6-*O*-methylCatalpol:
[H][C@@]12OC1(CO)[C@@]1([H])[C@H](OC3O[C@@H](CO)[C@H](O)[C@@H](O)[C@@H]3O)OC=C[C@@]1([H])[C@@H]2OC
Target PDB ID: 4e2b_modified.pdb
Box center: 23 - -5 - -1
Box size: 20 - 20 - 20

4E2B

High resolution crystal structure of the old yellow enzyme from Trypanosoma cruzi

PDB DOI: https://doi.org/10.2210/pdb4E2B/pdb

Classification: OXIDOREDUCTASE
Organism(s): Trypanosoma cruzi
Expression System: Escherichia coli
Mutation(s): No

Deposited: 2012-03-08 **Released:** 2013-03-27
Deposition Author(s): Murakami, M.T., Rodrigues, N.C., Gava, L.M., Canduri, F., Oliva, G., Barbosa, L.R.S., Borgers, J.C.

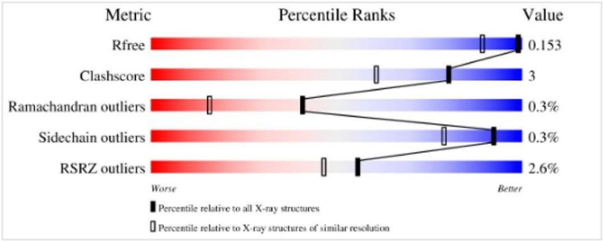

Figure S12. OYE protein obtained through the PDB database ID: 4E2B, and the docking parameters GRID box display from Swissdock software online.

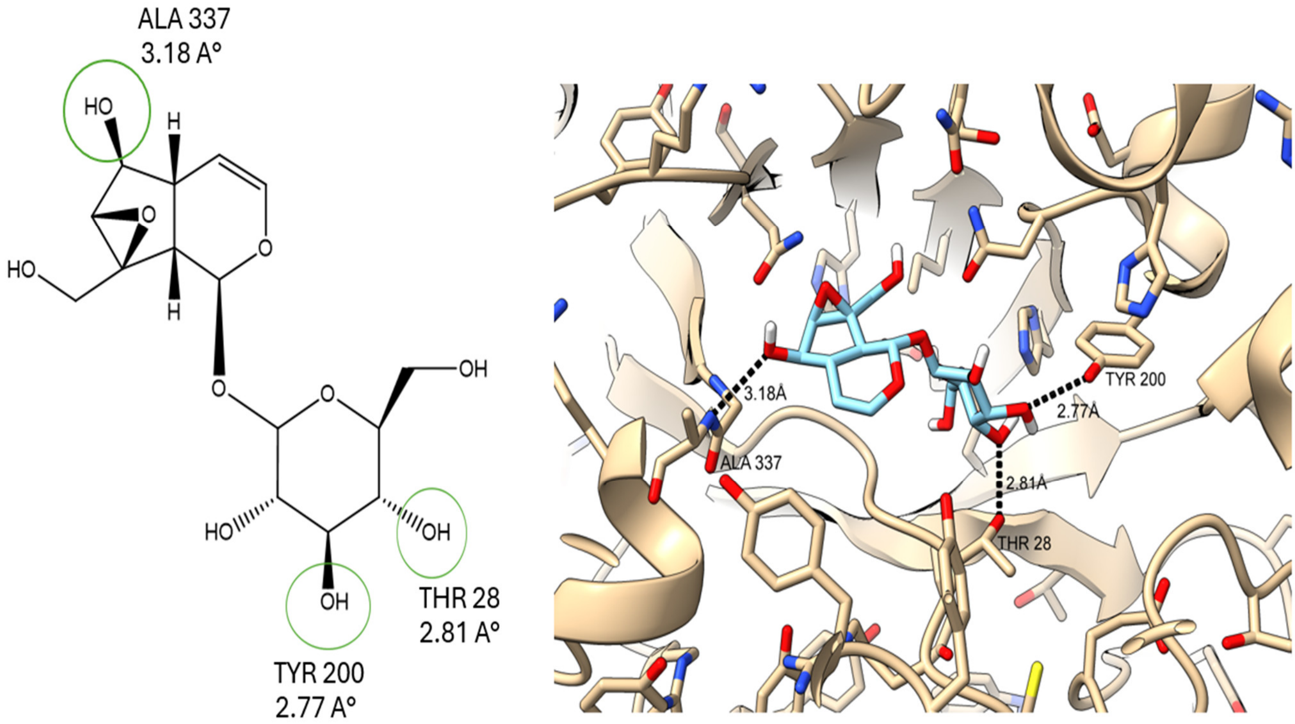

Figure S13. Molecular docking of catalpol with the OYE enzyme. The image illustrates hydrogen bond interactions between catalpol (light blue) and key residues of the OYE enzyme (beige ribbon). Specific hydrogen bond distances are shown: 2.81 Å with THR 28, 2.77 Å with TYR 200, and 3.18 Å with ALA 337.The binding energy for this interaction is -6.700 Kcal/mol.

Table S1. One-way ANOVA analysis of data from the TPC assay as indicated in Table 2, assuming a normal (Gaussian) distribution.

**ANOVA summary**	
F	484.0
P value	<0.0001
P value summary	****
Significant diff. among means (P < 0.05)?	Yes
R square	0.9954

**ANOVA table**	**SS**	**DF**	**MS**	**F (DFn. DFd)**	**P value**
Treatment (between columns)	6539325	8	817416	F (8. 18) = 484.0	P<0.0001
Residual (within columns)	30400	18	1689		
Total	6569725	26			

**Model comparison**	**SS**	**DF**	**Probability it is correct**
Null H. All population means identical	6569725	26	0%
Alternative H: Distinct population means	30400	18	100%
Ratio of probabilities			0.000
Difference in AICc			115.9

**Data summary**	
Number of treatments (columns)	9
Number of values (total)	27

Table S2. Tukey's multiple comparison test of data from the TPC assay as indicated in Table 2, assuming a normal (Gaussian) distribution.

Number of families	1
Number of comparisons per family	36
Alpha	0.05

Tukey's multiple comparisons test	Mean Diff.	95.00% CI of diff.	Significant?	Summary	Adjusted P Value	
BG200 vs. MET	-1570	-1687 to -1452	Yes	****	<0.0001	b-a
BG300 vs. MET	-952.2	-1070 to -834.6	Yes	****	<0.0001	c-a
(BG400) vs. MET	-578.6	-696.2 to -461.0	Yes	****	<0.0001	d-a
(BG500) vs. MET	-965.8	-1083 to -848.2	Yes	****	<0.0001	e-a
BG600 vs. MET	-389.8	-507.4 to -272.2	Yes	****	<0.0001	f-a
BG700 vs. MET	-1307	-1424 to -1189	Yes	****	<0.0001	g-a
BG800 vs. MET	-1401	-1519 to -1284	Yes	****	<0.0001	h-a
BG900 vs. MET	-1284	-1401 to -1166	Yes	****	<0.0001	i-a
BG300 vs. BG200	617.7	500.1 to 735.3	Yes	****	<0.0001	c-b
(BG400) vs. BG200	991.3	873.7 to 1109	Yes	****	<0.0001	d-b
(BG500) vs. BG200	604.1	486.5 to 721.7	Yes	****	<0.0001	e-b
BG600 vs. BG200	1180	1063 to 1298	Yes	****	<0.0001	f-b
BG700 vs. BG200	263.4	145.8 to 381.0	Yes	****	<0.0001	g-b
BG800 vs. BG200	168.5	50.93 to 286.1	Yes	**	0.0022	h-b
BG900 vs. BG200	286.3	168.7 to 403.9	Yes	****	<0.0001	i-b
(BG400) vs. BG300	373.6	256.0 to 491.2	Yes	****	<0.0001	d-c
(BG500) vs. BG300	-13.60	-131.2 to 104.0	No	ns	>0.9999	e-c
BG600 vs. BG300	562.4	444.8 to 680.0	Yes	****	<0.0001	f-c
BG700 vs. BG300	-354.3	-471.9 to -236.7	Yes	****	<0.0001	g-c
BG800 vs. BG300	-449.2	-566.8 to -331.6	Yes	****	<0.0001	h-c
BG900 vs. BG300	-331.4	-449.0 to -213.8	Yes	****	<0.0001	i-c
(BG500) vs. (BG400)	-387.2	-504.8 to -269.6	Yes	****	<0.0001	e-d
BG600 vs. (BG400)	188.8	71.23 to 306.4	Yes	***	0.0006	f-d
BG700 vs. (BG400)	-727.9	-845.5 to -610.3	Yes	****	<0.0001	g-d
BG800 vs. (BG400)	-822.8	-940.4 to -705.2	Yes	****	<0.0001	h-d
BG900 vs. (BG400)	-705.0	-822.6 to -587.4	Yes	****	<0.0001	i-d
BG600 vs. (BG500)	576.0	458.4 to 693.6	Yes	****	<0.0001	f-e
BG700 vs. (BG500)	-340.7	-458.3 to -223.1	Yes	****	<0.0001	g-e
BG800 vs. (BG500)	-435.6	-553.2 to -318.0	Yes	****	<0.0001	h-e
BG900 vs. (BG500)	-317.8	-435.4 to -200.2	Yes	****	<0.0001	i-e
BG700 vs. BG600	-916.7	-1034 to -799.1	Yes	****	<0.0001	g-f
BG800 vs. BG600	-1012	-1129 to -894.0	Yes	****	<0.0001	h-f
BG900 vs. BG600	-893.8	-1011 to -776.2	Yes	****	<0.0001	i-f
BG800 vs. BG700	-94.90	-212.5 to 22.67	No	ns	0.1736	h-g
BG900 vs. BG700	22.90	-94.67 to 140.5	No	ns	0.9984	i-g
BG900 vs. BG800	117.8	0.2286 to 235.4	Yes	*	0.0493	i-h

Test details	Mean 1	Mean 2	Mean Diff.	SE of diff.	n1	n2	q	DF
BG200 vs. MET	44.80	1615	-1570	33.55	3	3	66.17	18
BG300 vs. MET	662.5	1615	-952.2	33.55	3	3	40.13	18
(BG400) vs. MET	1036	1615	-578.6	33.55	3	3	24.39	18
(BG500) vs. MET	648.9	1615	-965.8	33.55	3	3	40.70	18
BG600 vs. MET	1225	1615	-389.8	33.55	3	3	16.43	18

BG700 vs. MET	308.2	1615	-1307	33.55	3	3	55.06	18
BG800 vs. MET	213.3	1615	-1401	33.55	3	3	59.06	18
BG900 vs. MET	331.1	1615	-1284	33.55	3	3	54.10	18
BG300 vs. BG200	662.5	44.80	617.7	33.55	3	3	26.03	18
(BG400) vs. BG200	1036	44.80	991.3	33.55	3	3	41.78	18
(BG500) vs. BG200	648.9	44.80	604.1	33.55	3	3	25.46	18
BG600 vs. BG200	1225	44.80	1180	33.55	3	3	49.74	18
BG700 vs. BG200	308.2	44.80	263.4	33.55	3	3	11.10	18
BG800 vs. BG200	213.3	44.80	168.5	33.55	3	3	7.102	18
BG900 vs. BG200	331.1	44.80	286.3	33.55	3	3	12.07	18
(BG400) vs. BG300	1036	662.5	373.6	33.55	3	3	15.75	18
(BG500) vs. BG300	648.9	662.5	-13.60	33.55	3	3	0.5732	18
BG600 vs. BG300	1225	662.5	562.4	33.55	3	3	23.70	18
BG700 vs. BG300	308.2	662.5	-354.3	33.55	3	3	14.93	18
BG800 vs. BG300	213.3	662.5	-449.2	33.55	3	3	18.93	18
BG900 vs. BG300	331.1	662.5	-331.4	33.55	3	3	13.97	18
(BG500) vs. (BG400)	648.9	1036	-387.2	33.55	3	3	16.32	18
BG600 vs. (BG400)	1225	1036	188.8	33.55	3	3	7.957	18
BG700 vs. (BG400)	308.2	1036	-727.9	33.55	3	3	30.68	18
BG800 vs. (BG400)	213.3	1036	-822.8	33.55	3	3	34.68	18
BG900 vs. (BG400)	331.1	1036	-705.0	33.55	3	3	29.71	18
BG600 vs. (BG500)	1225	648.9	576.0	33.55	3	3	24.28	18
BG700 vs. (BG500)	308.2	648.9	-340.7	33.55	3	3	14.36	18
BG800 vs. (BG500)	213.3	648.9	-435.6	33.55	3	3	18.36	18
BG900 vs. (BG500)	331.1	648.9	-317.8	33.55	3	3	13.39	18
BG700 vs. BG600	308.2	1225	-916.7	33.55	3	3	38.64	18
BG800 vs. BG600	213.3	1225	-1012	33.55	3	3	42.64	18
BG900 vs. BG600	331.1	1225	-893.8	33.55	3	3	37.67	18
BG800 vs. BG700	213.3	308.2	-94.90	33.55	3	3	4.000	18
BG900 vs. BG700	331.1	308.2	22.90	33.55	3	3	0.9652	18
BG900 vs. BG800	331.1	213.3	117.8	33.55	3	3	4.965	18

Table S3. One-way ANOVA analysis of data from the TFC assay as indicated in Table 2, assuming a normal (Gaussian) distribution.

**ANOVA summary**	
F	4701
P value	<0.0001
P value summary	****
Significant diff. among means (P < 0.05)?	Yes
R square	0.9995

**ANOVA table**	**SS**	**DF**	**MS**	**F (DFn, DFd)**	**P value**
Treatment (between columns)	668569	7	95510	F (7, 16) = 4701	P<0.0001
Residual (within columns)	325.0	16	20.32		
Total	668894	23			

**Model comparison**	**SS**	**DF**	**Probability it is correct**
Null H. All population means identical	668894	23	0%
Alternative H: Distinct population means	325.0	16	100%
Ratio of probabilities			0.000
Difference in AICc			156,8

**Data summary**	
Number of treatments (columns)	8
Number of values (total)	24

Table S4. Tukey's multiple comparison test test for the TFC assay as as indicated in Table 2, assuming a normal (Gaussian) distribution.

Number of families	1
Number of comparisons per family	28
Alpha	0,05

Tukey's multiple comparisons test	Mean Diff,	95,00% CI of diff,	Significant?	Summary	Adjusted P Value	
BG200 vs. MET	384.5	371.8 to 397.2	Yes	****	<0.0001	b-a
(BG500) vs. MET	-139.3	-152.0 to -126.6	Yes	****	<0.0001	c-a
BG600 vs. MET	-152.9	-165.6 to -140.2	Yes	****	<0.0001	d-a
BG700 vs. MET	-84.30	-97.04 to -71.56	Yes	****	<0.0001	e-a
BG800 vs. MET	-50.00	-62.74 to -37.26	Yes	****	<0.0001	f-a
BG900 vs. MET	-125.9	-138.6 to -113.2	Yes	****	<0.0001	g-a
BG1000 vs. MET	-135.1	-147.8 to -122.4	Yes	****	<0.0001	h-a
(BG500) vs. BG200	-523.8	-536.5 to -511.1	Yes	****	<0.0001	c-b
BG600 vs. BG200	-537.4	-550.1 to -524.7	Yes	****	<0.0001	d-b
BG700 vs. BG200	-468.8	-481.5 to -456.1	Yes	****	<0.0001	e-b
BG800 vs. BG200	-434.5	-447.2 to -421.8	Yes	****	<0.0001	f-b
BG900 vs. BG200	-510.4	-523.1 to -497.7	Yes	****	<0.0001	g-b
BG1000 vs. BG200	-519.6	-532.3 to -506.9	Yes	****	<0.0001	h-b
BG600 vs. (BG500)	-13.60	-26.34 to -0.8588	Yes	*	0.0322	d-c
BG700 vs. (BG500)	55.00	42.26 to 67.74	Yes	****	<0.0001	e-c
BG800 vs. (BG500)	89.30	76.56 to 102.0	Yes	****	<0.0001	f-c
BG900 vs. (BG500)	13.40	0.6588 to 26.14	Yes	*	0.0357	g-c
BG1000 vs. (BG500)	4.200	-8.541 to 16.94	No	ns	0.9373	h-c
BG700 vs. BG600	68.60	55.86 to 81.34	Yes	****	<0.0001	e-d
BG800 vs. BG600	102.9	90.16 to 115.6	Yes	****	<0.0001	f-d
BG900 vs. BG600	27.00	14.26 to 39.74	Yes	****	<0.0001	g-d
BG1000 vs. BG600	17.80	5.059 to 30.54	Yes	**	0.0035	h-d
BG800 vs. BG700	34.30	21.56 to 47.04	Yes	****	<0.0001	f-e
BG900 vs. BG700	-41.60	-54.34 to -28.86	Yes	****	<0.0001	g-e
BG1000 vs. BG700	-50.80	-63.54 to -38.06	Yes	****	<0.0001	h-e
BG900 vs. BG800	-75.90	-88.64 to -63.16	Yes	****	<0.0001	g-f
BG1000 vs. BG800	-85.10	-97.84 to -72.36	Yes	****	<0.0001	h-f
BG1000 vs. BG900	-9.200	-21.94 to 3.541	No	ns	0.2624	h-g

Test details	Mean 1	Mean 2	Mean Diff,	SE of diff,	n1	n2	q	DF
BG200 vs. MET	655.6	271.1	384.5	3.680	3	3	147.8	16
(BG500) vs. MET	131.8	271.1	-139.3	3.680	3	3	53.53	16
BG600 vs. MET	118.2	271.1	-152.9	3.680	3	3	58.76	16
BG700 vs. MET	186.8	271.1	-84.30	3.680	3	3	32.40	16
BG800 vs. MET	221.1	271.1	-50.00	3.680	3	3	19.21	16
BG900 vs. MET	145.2	271.1	-125.9	3.680	3	3	48.38	16
BG1000 vs. MET	136.0	271.1	-135.1	3.680	3	3	51.92	16
(BG500) vs. BG200	131.8	655.6	-523.8	3.680	3	3	201.3	16
BG600 vs. BG200	118.2	655.6	-537.4	3.680	3	3	206.5	16
BG700 vs. BG200	186.8	655.6	-468.8	3.680	3	3	180.2	16
BG800 vs. BG200	221.1	655.6	-434.5	3.680	3	3	167.0	16
BG900 vs. BG200	145.2	655.6	-510.4	3.680	3	3	196.1	16

BG1000 vs. BG200	136.0	655.6	-519.6	3.680	3	3	199.7	16
BG600 vs. (BG500)	118.2	131.8	-13.60	3.680	3	3	5.226	16
BG700 vs. (BG500)	186.8	131.8	55.00	3.680	3	3	21.14	16
BG800 vs. (BG500)	221.1	131.8	89.30	3.680	3	3	34.32	16
BG900 vs. (BG500)	145.2	131.8	13.40	3.680	3	3	5.149	16
BG1000 vs. (BG500)	136.0	131.8	4.200	3.680	3	3	1.614	16
BG700 vs. BG600	186.8	118.2	68.60	3.680	3	3	26.36	16
BG800 vs. BG600	221.1	118.2	102.9	3.680	3	3	39.54	16
BG900 vs. BG600	145.2	118.2	27.00	3.680	3	3	10.38	16
BG1000 vs. BG600	136.0	118.2	17.80	3.680	3	3	6,840	16
BG800 vs. BG700	221.1	186.8	34.30	3.680	3	3	13.18	16
BG900 vs. BG700	145.2	186.8	-41.60	3.680	3	3	15.99	16
BG1000 vs. BG700	136.0	186.8	-50.80	3.680	3	3	19.52	16
BG900 vs. BG800	145.2	221.1	-75.90	3.680	3	3	29.17	16
BG1000 vs. BG800	136.0	221.1	-85.10	3.680	3	3	32.70	16
BG1000 vs. BG900	136.0	145.2	-9.200	3.680	3	3	3,535	16

Table S5. One-way ANOVA analysis of data from the FRAP assay as indicated in Table 2, assuming a normal (Gaussian) distribution.

**ANOVA summary**					
F	1666				
P value	<0.0001				
P value summary	****				
Significant diff. among means (P < 0.05)?	Yes				
R square	0.9987				
**ANOVA table**	**SS**	**DF**	**MS**	**F (DFn. DFd)**	**P value**
Treatment (between columns)	2956232	10	295623	F (10. 22) = 1666	P<0.0001
Residual (within columns)	3903	22	177.4		
Total	2960134	32			
**Model comparison**	**SS**	**DF**	**Probability it is correct**		
Null H. All population means identical	2960134	32	0%		
Alternative H: Distinct population means	3903	22	100%		
Ratio of probabilities			0.000		
Difference in AICc			183.6		
**Data summary**					
Number of treatments (columns)	11				
Number of values (total)	33				

Table S6. Tukey's multiple comparison test analysis of data from the FRAP assay as indicated in Table 2, assuming a normal (Gaussian) distribution.

Number of families	1
Number of comparisons per family	55
Alpha	0.05

Tukey's multiple comparisons test	Mean Diff.	95.00% CI of diff.	Significant?	Summary	Adjusted P Value	
BG100 vs. MET	-975.3	-1014 to -936.4	Yes	****	<0.0001	b-a
BG200 vs. MET	-795.5	-834.4 to -756.6	Yes	****	<0.0001	c-a
BG300 vs. MET	-463.5	-502.4 to -424.6	Yes	****	<0.0001	d-a
(BG400) vs. MET	-118.5	-157.4 to -79.62	Yes	****	<0.0001	e-a
(BG500) vs. MET	-483.8	-522.7 to -444.9	Yes	****	<0.0001	f-a
BG600 vs. MET	-271.6	-310.5 to -232.7	Yes	****	<0.0001	g-a
BG700 vs. MET	-462.3	-501.2 to -423.4	Yes	****	<0.0001	h-a
BG800 vs. MET	-747.6	-786.5 to -708.7	Yes	****	<0.0001	i-a
BG900 vs. MET	-688.3	-727.2 to -649.4	Yes	****	<0.0001	j-a
BG1000 vs. MET	-871.0	-909.9 to -832.1	Yes	****	<0.0001	k-a
BG200 vs. BG100	179.8	140.9 to 218.7	Yes	****	<0.0001	c-b
BG300 vs. BG100	511.8	472.9 to 550.7	Yes	****	<0.0001	d-b
(BG400) vs. BG100	856.8	817.9 to 895.7	Yes	****	<0.0001	e-b
(BG500) vs. BG100	491.5	452.6 to 530.4	Yes	****	<0.0001	f-b
BG600 vs. BG100	703.7	664.8 to 742.6	Yes	****	<0.0001	g-b
BG700 vs. BG100	513.0	474.1 to 551.9	Yes	****	<0.0001	h-b
BG800 vs. BG100	227.7	188.8 to 266.6	Yes	****	<0.0001	i-b
BG900 vs. BG100	287.0	248.1 to 325.9	Yes	****	<0.0001	j-b
BG1000 vs. BG100	104.3	65.42 to 143.2	Yes	****	<0.0001	k-b
BG300 vs. BG200	332.0	293.1 to 370.9	Yes	****	<0.0001	d-c
(BG400) vs. BG200	677.0	638.1 to 715.9	Yes	****	<0.0001	e-c
(BG500) vs. BG200	311.7	272.8 to 350.6	Yes	****	<0.0001	f-c
BG600 vs. BG200	523.9	485.0 to 562.8	Yes	****	<0.0001	g-c
BG700 vs. BG200	333.2	294.3 to 372.1	Yes	****	<0.0001	h-c
BG800 vs. BG200	47.90	9.024 to 86.78	Yes	**	0.0081	i-c
BG900 vs. BG200	107.2	68.32 to 146.1	Yes	****	<0.0001	j-c
BG1000 vs. BG200	-75.50	-114.4 to -36.62	Yes	****	<0.0001	k-c
(BG400) vs. BG300	345.0	306.1 to 383.9	Yes	****	<0.0001	e-d
(BG500) vs. BG300	-20.30	-59.18 to 18.58	No	ns	0.7307	f-d
BG600 vs. BG300	191.9	153.0 to 230.8	Yes	****	<0.0001	g-d
BG700 vs. BG300	1.200	-37.68 to 40.08	No	ns	>0.9999	h-d
BG800 vs. BG300	-284.1	-323.0 to -245.2	Yes	****	<0.0001	i-d
BG900 vs. BG300	-224.8	-263.7 to -185.9	Yes	****	<0.0001	j-d
BG1000 vs. BG300	-407.5	-446.4 to -368.6	Yes	****	<0.0001	k-d
(BG500) vs. (BG400)	-365.3	-404.2 to -326.4	Yes	****	<0.0001	f-e
BG600 vs. (BG400)	-153.1	-192.0 to -114.2	Yes	****	<0.0001	g-e
BG700 vs. (BG400)	-343.8	-382.7 to -304.9	Yes	****	<0.0001	h-e
BG800 vs. (BG400)	-629.1	-668.0 to -590.2	Yes	****	<0.0001	i-e
BG900 vs. (BG400)	-569.8	-608.7 to -530.9	Yes	****	<0.0001	j-e
BG1000 vs. (BG400)	-752.5	-791.4 to -713.6	Yes	****	<0.0001	k-e
BG600 vs. (BG500)	212.2	173.3 to 251.1	Yes	****	<0.0001	g-f
BG700 vs. (BG500)	21.50	-17.38 to 60.38	No	ns	0.6650	h-f

BG800 vs. (BG500)	-263.8	-302.7 to -224.9	Yes	****	<0.0001	i-f
BG900 vs. (BG500)	-204.5	-243.4 to -165.6	Yes	****	<0.0001	j-f
BG1000 vs. (BG500)	-387.2	-426.1 to -348.3	Yes	****	<0.0001	k-f
BG700 vs. BG600	-190.7	-229.6 to -151.8	Yes	****	<0.0001	h-g
BG800 vs. BG600	-476.0	-514.9 to -437.1	Yes	****	<0.0001	i-g
BG900 vs. BG600	-416.7	-455.6 to -377.8	Yes	****	<0.0001	j-g
BG1000 vs. BG600	-599.4	-638.3 to -560.5	Yes	****	<0.0001	k-g
BG800 vs. BG700	-285.3	-324.2 to -246.4	Yes	****	<0.0001	i-h
BG900 vs. BG700	-226.0	-264.9 to -187.1	Yes	****	<0.0001	j-h
BG1000 vs. BG700	-408.7	-447.6 to -369.8	Yes	****	<0.0001	k-h
BG900 vs. BG800	59.30	20.42 to 98.18	Yes	***	0.0007	j-i
BG1000 vs. BG800	-123.4	-162.3 to -84.52	Yes	****	<0.0001	k-i
BG1000 vs. BG900	-182.7	-221.6 to -143.8	Yes	****	<0.0001	k-j

**Test details**	**Mean 1**	**Mean 2**	**Mean Diff.**	**SE of diff.**	**n1**	**n2**	**q**	**DF**
BG100 vs. MET	46.20	1022	-975.3	10.87	3	3	126.8	22
BG200 vs. MET	226.0	1022	-795.5	10.87	3	3	103.4	22
BG300 vs. MET	558.0	1022	-463.5	10.87	3	3	60.27	22
(BG400) vs. MET	903.0	1022	-118.5	10.87	3	3	15.41	22
(BG500) vs. MET	537.7	1022	-483.8	10.87	3	3	62.91	22
BG600 vs. MET	749.9	1022	-271.6	10.87	3	3	35.32	22
BG700 vs. MET	559.2	1022	-462.3	10.87	3	3	60.12	22
BG800 vs. MET	273.9	1022	-747.6	10.87	3	3	97.22	22
BG900 vs. MET	333.2	1022	-688.3	10.87	3	3	89.51	22
BG1000 vs. MET	150.5	1022	-871.0	10.87	3	3	113.3	22
BG200 vs. BG100	226.0	46.20	179.8	10.87	3	3	23.38	22
BG300 vs. BG100	558.0	46.20	511.8	10.87	3	3	66.56	22
(BG400) vs. BG100	903.0	46.20	856.8	10.87	3	3	111.4	22
(BG500) vs. BG100	537.7	46.20	491.5	10.87	3	3	63.92	22
BG600 vs. BG100	749.9	46.20	703.7	10.87	3	3	91.51	22
BG700 vs. BG100	559.2	46.20	513.0	10.87	3	3	66.71	22
BG800 vs. BG100	273.9	46.20	227.7	10.87	3	3	29.61	22
BG900 vs. BG100	333.2	46.20	287.0	10.87	3	3	37.32	22
BG1000 vs. BG100	150.5	46.20	104.3	10.87	3	3	13.56	22
BG300 vs. BG200	558.0	226.0	332.0	10.87	3	3	43.17	22
(BG400) vs. BG200	903.0	226.0	677.0	10.87	3	3	88.04	22
(BG500) vs. BG200	537.7	226.0	311.7	10.87	3	3	40.53	22
BG600 vs. BG200	749.9	226.0	523.9	10.87	3	3	68.13	22
BG700 vs. BG200	559.2	226.0	333.2	10.87	3	3	43.33	22
BG800 vs. BG200	273.9	226.0	47.90	10.87	3	3	6.229	22
BG900 vs. BG200	333.2	226.0	107.2	10.87	3	3	13.94	22
BG1000 vs. BG200	150.5	226.0	-75.50	10.87	3	3	9.818	22
(BG400) vs. BG300	903.0	558.0	345.0	10.87	3	3	44.86	22
(BG500) vs. BG300	537.7	558.0	-20.30	10.87	3	3	2.640	22
BG600 vs. BG300	749.9	558.0	191.9	10.87	3	3	24.96	22
BG700 vs. BG300	559.2	558.0	1.200	10.87	3	3	0.1561	22
BG800 vs. BG300	273.9	558.0	-284.1	10.87	3	3	36.95	22
BG900 vs. BG300	333.2	558.0	-224.8	10.87	3	3	29.23	22
BG1000 vs. BG300	150.5	558.0	-407.5	10.87	3	3	52.99	22
(BG500) vs. (BG400)	537.7	903.0	-365.3	10.87	3	3	47.50	22
BG600 vs. (BG400)	749.9	903.0	-153.1	10.87	3	3	19.91	22

BG700 vs. (BG400)	559.2	903.0	-343.8	10.87	3	3	44.71	22
BG800 vs. (BG400)	273.9	903.0	-629.1	10.87	3	3	81.81	22
BG900 vs. (BG400)	333.2	903.0	-569.8	10.87	3	3	74.10	22
BG1000 vs. (BG400)	150.5	903.0	-752.5	10.87	3	3	97.86	22
BG600 vs. (BG500)	749.9	537.7	212.2	10.87	3	3	27.60	22
BG700 vs. (BG500)	559.2	537.7	21.50	10.87	3	3	2.796	22
BG800 vs. (BG500)	273.9	537.7	-263.8	10.87	3	3	34.31	22
BG900 vs. (BG500)	333.2	537.7	-204.5	10.87	3	3	26.59	22
BG1000 vs. (BG500)	150.5	537.7	-387.2	10.87	3	3	50.35	22
BG700 vs. BG600	559.2	749.9	-190.7	10.87	3	3	24.80	22
BG800 vs. BG600	273.9	749.9	-476.0	10.87	3	3	61.90	22
BG900 vs. BG600	333.2	749.9	-416.7	10.87	3	3	54.19	22
BG1000 vs. BG600	150.5	749.9	-599.4	10.87	3	3	77.95	22
BG800 vs. BG700	273.9	559.2	-285.3	10.87	3	3	37.10	22
BG900 vs. BG700	333.2	559.2	-226.0	10.87	3	3	29.39	22
BG1000 vs. BG700	150.5	559.2	-408.7	10.87	3	3	53.15	22
BG900 vs. BG800	333.2	273.9	59.30	10.87	3	3	7.712	22
BG1000 vs. BG800	150.5	273.9	-123.4	10.87	3	3	16.05	22
BG1000 vs. BG900	150.5	333.2	-182.7	10.87	3	3	23.76	22

Table S7. One-way ANOVA analysis of data from the ABTS+ assay as indicated in Table 2, assuming a normal (Gaussian) distribution.

**ANOVA summary**	
F	1003
P value	<0.0001
P value summary	****
Significant diff. among means (P < 0.05)?	Yes
R square	0.9978

**ANOVA table**	**SS**	**DF**	**MS**	**F (DFn. DFd)**	**P value**
Treatment (between columns)	2271758	8	283970	F (8. 18) = 1003	P<0.0001
Residual (within columns)	5095	18	283.1		
Total	2276853	26			

**Model comparison**	**SS**	**DF**	**Probability it is correct**
Null H. All population means identical	2276853	26	0%
Alternative H: Distinct population means	5095	18	100%
Ratio of probabilities			0.000
Difference in AICc			135.5

**Data summary**	
Number of treatments (columns)	9
Number of values (total)	27

Table S8. Tukey's multiple comparison test analysis of data from the ABTS+ assay as indicated in Table 2, assuming a normal (Gaussian) distribution.

Number of families	1
Number of comparisons per family	36
Alpha	0,05

Tukey's multiple comparisons test	Mean Diff,	95,00% CI of diff,	Significant?	Summary	Adjusted P Value	
BG300 vs. MET	-343.8	-391.9 to -295.7	Yes	****	<0.0001	b-a
(BG400) vs. MET	119.2	71.07 to 167.3	Yes	****	<0.0001	c-a
(BG500) vs. MET	-467.7	-515.8 to -419.6	Yes	****	<0.0001	d-a
BG600 vs. MET	-331.7	-379.8 to -283.6	Yes	****	<0.0001	e-a
BG700 vs. MET	-636.6	-684.7 to -588.5	Yes	****	<0.0001	f-a
BG800 vs. MET	-650.4	-698.5 to -602.3	Yes	****	<0.0001	g-a
BG900 vs. MET	-654.8	-702.9 to -606.7	Yes	****	<0.0001	h-a
BG1000 vs. MET	-765.5	-813.6 to -717.4	Yes	****	<0.0001	i-a
(BG400) vs. BG300	463.0	414.9 to 511.1	Yes	****	<0.0001	c-b
(BG500) vs. BG300	-123.9	-172.0 to -75.77	Yes	****	<0.0001	d-b
BG600 vs. BG300	12.10	-36.03 to 60.23	No	ns	0.9914	e-b
BG700 vs. BG300	-292.8	-340.9 to -244.7	Yes	****	<0.0001	f-b
BG800 vs. BG300	-306.6	-354.7 to -258.5	Yes	****	<0.0001	g-b
BG900 vs. BG300	-311.0	-359.1 to -262.9	Yes	****	<0.0001	h-b
BG1000 vs. BG300	-421.7	-469.8 to -373.6	Yes	****	<0.0001	i-b
(BG500) vs. (BG400)	-586.9	-635.0 to -538.8	Yes	****	<0.0001	d-c
BG600 vs. (BG400)	-450.9	-499.0 to -402.8	Yes	****	<0.0001	e-c
BG700 vs. (BG400)	-755.8	-803.9 to -707.7	Yes	****	<0.0001	f-c
BG800 vs. (BG400)	-769.6	-817.7 to -721.5	Yes	****	<0.0001	g-c
BG900 vs. (BG400)	-774.0	-822.1 to -725.9	Yes	****	<0.0001	h-c
BG1000 vs. (BG400)	-884.7	-932.8 to -836.6	Yes	****	<0.0001	i-c
BG600 vs. (BG500)	136.0	87.87 to 184.1	Yes	****	<0.0001	e-d
BG700 vs. (BG500)	-168.9	-217.0 to -120.8	Yes	****	<0.0001	f-d
BG800 vs. (BG500)	-182.7	-230.8 to -134.6	Yes	****	<0.0001	g-d
BG900 vs. (BG500)	-187.1	-235.2 to -139.0	Yes	****	<0.0001	h-d
BG1000 vs. (BG500)	-297.8	-345.9 to -249.7	Yes	****	<0.0001	i-d
BG700 vs. BG600	-304.9	-353.0 to -256.8	Yes	****	<0.0001	f-e
BG800 vs. BG600	-318.7	-366.8 to -270.6	Yes	****	<0.0001	g-e
BG900 vs. BG600	-323.1	-371.2 to -275.0	Yes	****	<0.0001	h-e
BG1000 vs. BG600	-433.8	-481.9 to -385.7	Yes	****	<0.0001	i-e
BG800 vs. BG700	-13.80	-61.93 to 34.33	No	ns	0.9805	g-f
BG900 vs. BG700	-18.20	-66.33 to 29.93	No	ns	0.9108	h-f
BG1000 vs. BG700	-128.9	-177.0 to -80.77	Yes	****	<0.0001	i-f
BG900 vs. BG800	-4.400	-52.53 to 43.73	No	ns	>0.9999	h-g
BG1000 vs. BG800	-115.1	-163.2 to -66.97	Yes	****	<0.0001	i-g
BG1000 vs. BG900	-110.7	-158.8 to -62.57	Yes	****	<0.0001	i-h

Test details	Mean 1	Mean 2	Mean Diff.	SE of diff.	n1	n2	q	DF
BG300 vs. MET	505.2	849.0	-343.8	13.74	3	3	35.39	18
(BG400) vs. MET	968.2	849.0	119.2	13.74	3	3	12.27	18
(BG500) vs. MET	381.3	849.0	-467.7	13.74	3	3	48.15	18
BG600 vs. MET	517.3	849.0	-331.7	13.74	3	3	34.15	18

BG700 vs. MET	212.4	849.0	-636.6	13.74	3	3	65.54	18
BG800 vs. MET	198.6	849.0	-650.4	13.74	3	3	66.96	18
BG900 vs. MET	194.2	849.0	-654.8	13.74	3	3	67.41	18
BG1000 vs. MET	83.50	849.0	-765.5	13.74	3	3	78.81	18
(BG400) vs. BG300	968.2	505.2	463.0	13.74	3	3	47.67	18
(BG500) vs. BG300	381.3	505.2	-123.9	13.74	3	3	12.76	18
BG600 vs. BG300	517.3	505.2	12.10	13.74	3	3	1.246	18
BG700 vs. BG300	212.4	505.2	-292.8	13.74	3	3	30.14	18
BG800 vs. BG300	198.6	505.2	-306.6	13.74	3	3	31.56	18
BG900 vs. BG300	194.2	505.2	-311.0	13.74	3	3	32.02	18
BG1000 vs. BG300	83.50	505.2	-421.7	13.74	3	3	43.41	18
(BG500) vs. (BG400)	381.3	968.2	-586.9	13.74	3	3	60.42	18
BG600 vs. (BG400)	517.3	968.2	-450.9	13.74	3	3	46.42	18
BG700 vs. (BG400)	212.4	968.2	-755.8	13.74	3	3	77.81	18
BG800 vs. (BG400)	198.6	968.2	-769.6	13.74	3	3	79.23	18
BG900 vs. (BG400)	194.2	968.2	-774.0	13.74	3	3	79.68	18
BG1000 vs. (BG400)	83.50	968.2	-884.7	13.74	3	3	91.08	18
BG600 vs. (BG500)	517.3	381.3	136.0	13.74	3	3	14.00	18
BG700 vs. (BG500)	212.4	381.3	-168.9	13.74	3	3	17.39	18
BG800 vs. (BG500)	198.6	381.3	-182.7	13.74	3	3	18.81	18
BG900 vs. (BG500)	194.2	381.3	-187.1	13.74	3	3	19.26	18
BG1000 vs. (BG500)	83.50	381.3	-297.8	13.74	3	3	30.66	18
BG700 vs. BG600	212.4	517.3	-304.9	13.74	3	3	31.39	18
BG800 vs. BG600	198.6	517.3	-318.7	13.74	3	3	32.81	18
BG900 vs. BG600	194.2	517.3	-323.1	13.74	3	3	33.26	18
BG1000 vs. BG600	83.50	517.3	-433.8	13.74	3	3	44.66	18
BG800 vs. BG700	198.6	212.4	-13.80	13.74	3	3	1.421	18
BG900 vs. BG700	194.2	212.4	-18.20	13.74	3	3	1.874	18
BG1000 vs. BG700	83.50	212.4	-128.9	13.74	3	3	13.27	18
BG900 vs. BG800	194.2	198.6	-4.400	13.74	3	3	0.4530	18
BG1000 vs. BG800	83.50	198.6	-115.1	13.74	3	3	11.85	18
BG1000 vs. BG900	83.50	194.2	-110.7	13.74	3	3	11.40	18

Table S9. One-way ANOVA analysis of data from the DPPH assay as indicated in Table 2, assuming a normal (Gaussian) distribution.

**ANOVA summary**	
F	1127
P value	<0.0001
P value summary	****
Significant diff. among means (P < 0.05)?	Yes
R square	0.9980

**ANOVA table**	**SS**	**DF**	**MS**	**F (DFn, DFd)**	**P value**
Treatment (between columns)	1310496	7	187214	F (7, 16) = 1127	P<0.0001
Residual (within columns)	2657	16	166.1		
Total	1313153	23			

**Model comparison**	**SS**	**DF**	**Probability it is correct**
Null H. All population means identical	1313153	23	0%
Alternative H: Distinct population means	2657	16	100%
Ratio of probabilities			0.000
Difference in AICc			122.6

**Data summary**	
Number of treatments (columns)	8
Number of values (total)	24

Table S10. Tukey's multiple comparison test analysis of data from the DPPH assay as indicated in Table 2, assuming a normal (Gaussian) distribution.

Number of families	1
Number of comparisons per family	28
Alpha	0,05

Tukey's multiple comparisons test	Mean Diff,	95,00% CI of diff,	Significant?	Summary	Adjusted P Value	
BG300 vs. MET	-590.2	-626.6 to -553.8	Yes	****	<0.0001	b-a
(BG400) vs. MET	-418.7	-455.1 to -382.3	Yes	****	<0.0001	c-a
(BG500) vs. MET	-537.0	-573.4 to -500.6	Yes	****	<0.0001	d-a
BG600 vs. MET	-244.4	-280.8 to -208.0	Yes	****	<0.0001	e-a
BG700 vs. MET	-522.8	-559.2 to -486.4	Yes	****	<0.0001	f-a
BG800 vs. MET	-739.4	-775.8 to -703.0	Yes	****	<0.0001	g-a
BG900 vs. MET	-731.1	-767.5 to -694.7	Yes	****	<0.0001	h-a
(BG400) vs. BG300	171.5	135.1 to 207.9	Yes	****	<0.0001	c-b
(BG500) vs. BG300	53.20	16.77 to 89.63	Yes	**	0.0023	d-b
BG600 vs. BG300	345.8	309.4 to 382.2	Yes	****	<0.0001	e-b
BG700 vs. BG300	67.40	30.97 to 103.8	Yes	***	0.0002	f-b
BG800 vs. BG300	-149.2	-185.6 to -112.8	Yes	****	<0.0001	g-b
BG900 vs. BG300	-140.9	-177.3 to -104.5	Yes	****	<0.0001	h-b
(BG500) vs. (BG400)	-118.3	-154.7 to -81.87	Yes	****	<0.0001	d-c
BG600 vs. (BG400)	174.3	137.9 to 210.7	Yes	****	<0.0001	e-c
BG700 vs. (BG400)	-104.1	-140.5 to -67.67	Yes	****	<0.0001	f-c
BG800 vs. (BG400)	-320.7	-357.1 to -284.3	Yes	****	<0.0001	g-c
BG900 vs. (BG400)	-312.4	-348.8 to -276.0	Yes	****	<0.0001	h-c
BG600 vs. (BG500)	292.6	256.2 to 329.0	Yes	****	<0.0001	e-d
BG700 vs. (BG500)	14.20	-22.23 to 50.63	No	ns	0.8665	f-d
BG800 vs. (BG500)	-202.4	-238.8 to -166.0	Yes	****	<0.0001	g-d
BG900 vs. (BG500)	-194.1	-230.5 to -157.7	Yes	****	<0.0001	h-d
BG700 vs. BG600	-278.4	-314.8 to -242.0	Yes	****	<0.0001	f-e
BG800 vs. BG600	-495.0	-531.4 to -458.6	Yes	****	<0.0001	g-e
BG900 vs. BG600	-486.7	-523.1 to -450.3	Yes	****	<0.0001	h-e
BG800 vs. BG700	-216.6	-253.0 to -180.2	Yes	****	<0.0001	g-f
BG900 vs. BG700	-208.3	-244.7 to -171.9	Yes	****	<0.0001	h-f
BG900 vs. BG800	8.300	-28.13 to 44.73	No	ns	0.9914	h-g

Test details	Mean 1	Mean 2	Mean Diff.	SE of diff.	n1	n2	q	DF
BG300 vs. MET	259.5	849.7	-590.2	10.52	3	3	79.32	16
(BG400) vs. MET	431.0	849.7	-418.7	10.52	3	3	56.27	16
(BG500) vs. MET	312.7	849.7	-537.0	10.52	3	3	72.17	16
BG600 vs. MET	605.3	849.7	-244.4	10.52	3	3	32.85	16
BG700 vs. MET	326.9	849.7	-522.8	10.52	3	3	70.26	16
BG800 vs. MET	110.3	849.7	-739.4	10.52	3	3	99.37	16
BG900 vs. MET	118.6	849.7	-731.1	10.52	3	3	98.26	16
(BG400) vs. BG300	431.0	259.5	171.5	10.52	3	3	23.05	16
(BG500) vs. BG300	312.7	259.5	53.20	10.52	3	3	7.150	16
BG600 vs. BG300	605.3	259.5	345.8	10.52	3	3	46.47	16
BG700 vs. BG300	326.9	259.5	67.40	10.52	3	3	9.058	16
BG800 vs. BG300	110.3	259.5	-149.2	10.52	3	3	20.05	16

BG900 vs. BG300	118.6	259.5	-140.9	10.52	3	3	18.94	16
(BG500) vs. (BG400)	312.7	431.0	-118.3	10.52	3	3	15.90	16
BG600 vs. (BG400)	605.3	431.0	174.3	10.52	3	3	23.43	16
BG700 vs. (BG400)	326.9	431.0	-104.1	10.52	3	3	13.99	16
BG800 vs. (BG400)	110.3	431.0	-320.7	10.52	3	3	43.10	16
BG900 vs. (BG400)	118.6	431.0	-312.4	10.52	3	3	41.99	16
BG600 vs. (BG500)	605.3	312.7	292.6	10.52	3	3	39.32	16
BG700 vs. (BG500)	326.9	312.7	14.20	10.52	3	3	1.908	16
BG800 vs. (BG500)	110.3	312.7	-202.4	10.52	3	3	27.20	16
BG900 vs. (BG500)	118.6	312.7	-194.1	10.52	3	3	26.09	16
BG700 vs. BG600	326.9	605.3	-278.4	10.52	3	3	37.42	16
BG800 vs. BG600	110.3	605.3	-495.0	10.52	3	3	66.53	16
BG900 vs. BG600	118.6	605.3	-486.7	10.52	3	3	65.41	16
BG800 vs. BG700	110.3	326.9	-216.6	10.52	3	3	29.11	16
BG900 vs. BG700	118.6	326.9	-208.3	10.52	3	3	27.99	16
BG900 vs. BG800	118.6	110.3	8.300	10.52	3	3	1.115	16

Table S11. Effect size estimation analysis (Time Factor) of MET, BG500 fraction and catalpol on the *T. cruzi* mitochondrial membrane potential. Two-way ANOVA analysis (Alpha=0,05).

Source of Variation	% of total variation	P value	P value summary	Significant?
Interaction	29.03	<0.0001	****	Yes
Time Factor	6.694	<0.0001	****	Yes
Treatment (concentration)	63.42	<0.0001	****	Yes

ANOVA table	SS	DF	MS	F (DFn. DFd)	P value
Interaction	50978	30	1699	F (30.88) = 98.63	P<0.0001
Time Factor	11758	3	3919	F (3.88) = 227.5	P<0.0001
Treatment (concentration)	111382	10	11138	F (10.88) = 646.5	P<0.0001
Residual	1516	88	17.23		

Data summary	
Number of columns (Treatment (concentration))	11
Number of rows (Time Factor)	4
Number of values	132

Dunnett's multiple comparisons test

	Mean diff.	95.00% CI of diff.	Below threshold?	Summary	Adjusted P Value
Untreated					
0 min vs. 30 min	0.11	-7.991 to 8.211	No	ns	>0.9999
0 min vs. 60 min	0.11	-7.991 to 8.211	No	ns	>0.9999
0 min vs. 120 min	0.11	-7.991 to 8.211	No	ns	>0.9999
CCCP (5.5 μg/mL)					
0 min vs. 30 min	20.11	12.01 to 28.21	Yes	****	<0.0001
0 min vs. 60 min	30.11	22.01 to 38.21	Yes	****	<0.0001
0 min vs. 120 min	44.11	36.01 to 52.21	Yes	****	<0.0001
B. globosa MET (500 μg/mL)					
0 min vs. 30 min	-53.89	-61.99 to -45.79	Yes	****	<0.0001
0 min vs. 60 min	-69.89	-77.99 to -61.79	Yes	****	<0.0001
0 min vs. 120 min	-70.89	-78.99 to -62.79	Yes	****	<0.0001
B. globosa MET (1000 μg/mL)					
0 min vs. 30 min	-100.9	-109.0 to -92.79	Yes	****	<0.0001
0 min vs. 60 min	-71.89	-79.99 to -63.79	Yes	****	<0.0001
0 min vs. 120 min	-53.89	-61.99 to -45.79	Yes	****	<0.0001
B. globosa MET (2000 μg/mL)					
0 min vs. 30 min	-103.9	-112.0 to -95.79	Yes	****	<0.0001
0 min vs. 60 min	-129.9	-138.0 to -121.8	Yes	****	<0.0001
0 min vs. 120 min	-69.89	-77.99 to -61.79	Yes	****	<0.0001
BG500 (500 μg/mL)					

0 min vs. 30 min	2.11	-5.991 to 10.21	No	ns	0.8681
0 min vs. 60 min	9.11	1.009 to 17.21	Yes	*	0.0234
0 min vs. 120 min	10.11	2.009 to 18.21	Yes	*	0.0103
BG500 (1000 µg/mL)					
0 min vs. 30 min	-16.89	-24.99 to -8.789	Yes	****	<0.0001
0 min vs. 60 min	11.11	3.009 to 19.21	Yes	**	0.0042
0 min vs. 120 min	8.11	0.008925 to 16.21	Yes	*	0.0497
BG500 (2000 µg/mL)					
0 min vs. 30 min	-17.89	-25.99 to -9.789	Yes	****	<0.0001
0 min vs. 60 min	-2.89	-10.99 to 5.211	No	ns	0.7278
0 min vs. 120 min	-11.89	-19.99 to -3.789	Yes	**	0.002
Catalpol (72.4 µg/mL)					
0 min vs. 30 min	3.11	-4.991 to 11.21	No	ns	0.6835
0 min vs. 60 min	-6.89	-14.99 to 1.211	No	ns	0.1132
0 min vs. 120 min	-23.89	-31.99 to -15.79	Yes	****	<0.0001
Catalpol (144.9 µg/mL)					
0 min vs. 30 min	4.11	-3.991 to 12.21	No	ns	0.4811
0 min vs. 60 min	-7.89	-15.99 to 0.2111	No	ns	0.0581
0 min vs. 120 min	-18.89	-26.99 to -10.79	Yes	****	<0.0001
Catalpol (289.8 µg/mL)					
0 min vs. 30 min	4.11	-3.991 to 12.21	No	ns	0.4811
0 min vs. 60 min	-4.89	-12.99 to 3.211	No	ns	0.342
0 min vs. 120 min	-11.89	-19.99 to -3.789	Yes	**	0.002

Table S12. Effect size estimation analysis (Concentration Factor) of the MET, BG500 fraction and catalpol on the *T. cruzi* mitochondrial membrane potential. Two-way ANOVA analysis (Alpha=0.05).

Source of Variation	% of total variation	P value	P value summary	Significant?
Interaction	8.424	<0.0001	****	Yes
Row Factor	90.32	<0.0001	****	Yes
Column Factor	0.3319	<0.0001	****	Yes

ANOVA table	SS	DF	MS	F (DFn, DFd)	P value
Interaction	13850	20	692,5	F (20, 66) = 30,18	P<0,0001
Row Factor	148510	10	14851	F (10, 66) = 647,3	P<0,0001
Column Factor	545,6	2	272,8	F (2, 66) = 11,89	P<0,0001
Residual	1514	66	22,94		

Data summary	
Number of columns (Column Factor)	3
Number of rows (Row Factor)	11
Number of values	99

Dunnett's multiple comparisons test

	Mean dif.	95.00% CI of diff.	Below threshold?	Summary	Adjusted P Value
30 min					
Parasites vs. CCCP (5.5 μg/mL)	20.00	9.075 to 30.92	Yes	****	<0.0001
Parasites vs. MET (500 μg/mL)	-54.00	-64.92 to -43.08	Yes	****	<0.0001
Parasites vs. MET (1000 μg/mL)	-101.0	-111.9 to -90.08	Yes	****	<0.0001
Parasites vs. MET (2000 μg/mL)	-104.0	-114.9 to -93.08	Yes	****	<0.0001
Parasites vs. BG500 (500 μg/mL)	2.000	-8.925 to 12.92	No	ns	0.9993
Parasites vs. BG500 (1000 μg/mL)	-17.00	-27.92 to -6.075	Yes	***	0.0004
Parasites vs. BG500 (2000 μg/mL)	-18.00	-28.92 to -7.075	Yes	***	0.0002
Parasites vs. Catalpol (72.4 μg/mL)	3.000	-7.925 to 13.92	No	ns	0.9846
Parasites vs. Catalpol (144.9 μg/mL)	4.000	-6.925 to 14.92	No	ns	0.9133
Parasites vs. Catalpol (289.8 μg/mL)	4.000	-6.925 to 14.92	No	ns	0.9133
60 min					
Parasites vs. CCCP (5.5 μg/mL)	30.00	19.08 to 40.92	Yes	****	<0.0001
Parasites vs. MET (500 μg/mL)	-70.00	-80.92 to -59.08	Yes	****	<0.0001
Parasites vs. MET (1000 μg/mL)	-72.00	-82.92 to -61.08	Yes	****	<0.0001
Parasites vs. MET (2000 μg/mL)	-130.0	-140.9 to -119.1	Yes	****	<0.0001
Parasites vs. BG500 (500 μg/mL)	9.000	-1.925 to 19.92	No	ns	0.1554
Parasites vs. BG500 (1000 μg/mL)	11.00	0.07505 to 21.92	Yes	*	0.0476
Parasites vs. BG500 (2000 μg/mL)	-3.000	-13.92 to 7.925	No	ns	0.9846
Parasites vs. Catalpol (72.4 μg/mL)	-7.000	-17.92 to 3.925	No	ns	0.3998
Parasites vs. Catalpol (144.9 μg/mL)	-8.000	-18.92 to 2.925	No	ns	0.2574
Parasites vs. Catalpol (289.8 μg/mL)	-5.000	-15.92 to 5.925	No	ns	0.7651

120 min					
Parasites vs. CCCP (5.5 µg/mL)	44.00	33.08 to 54.92	Yes	****	<0.0001
Parasites vs. MET (500 µg/mL)	-71.00	-81.92 to -60.08	Yes	****	<0.0001
Parasites vs. MET (1000 µg/mL)	-54.00	-64.92 to -43.08	Yes	****	<0.0001
Parasites vs. MET (2000 µg/mL)	-70.00	-80.92 to -59.08	Yes	****	<0.0001
Parasites vs. BG500 (500 µg/mL)	10.00	-0.9249 to 20.92	No	ns	0.0884
Parasites vs. BG500 (1000 µg/mL)	8.000	-2.925 to 18.92	No	ns	0.2574
Parasites vs. BG500 (2000 µg/mL)	-12.00	-22.92 to -1.075	Yes	*	0.0244
Parasites vs. Catalpol (72.4 µg/mL)	-24.00	-34.92 to -13.08	Yes	****	<0.0001
Parasites vs. Catalpol (144.9 µg/mL)	-19.00	-29.92 to -8.075	Yes	****	<0.0001
Parasites vs. Catalpol (289.8 µg/mL)	-12.00	-22.92 to -1.075	Yes	*	0.0244

Table S13. AutoDock Vina results showing the predicted binding energies (kcal/mol) for various conformations (models) of FMN and 6-*O*-mehylcatalpol bound to OYE. The models represent different orientations of each ligand within the active site.

**FMN**		**6-*O*-methylcatalpol**	
Model	Calculated Afinity (kcal/mol)	Model	Calculated Afinity (kcal/mol)
1	-7,000	1	-6.411
2	-6.921	2	-6.306
3	-6.786	3	-6.153
4	-6.645	4	-6.071
5	-6.643	5	-6.061
6	-6.559	6	-6.058
7	-6.541	7	-6.028
8	-6.295	8	-5.818
9	-6.195	9	-5.762
10	-6.088	10	-5.761
11	-6.081	11	-5.748
12	-5.938	12	-5.626
13	-5.853	13	-5.584
14	-5.277	14	-5.535
15	-4.497	15	-5.380
16	-4.432	16	-5.344
17	-4.355	17	-5.011
18	-4.021	18	-4.978
		19	-4.711
		20	-4.637
